# An occluded cherry tomato recognition model based on improved YOLOv7

**DOI:** 10.3389/fpls.2023.1260808

**Published:** 2023-10-20

**Authors:** Guangyu Hou, Haihua Chen, Yike Ma, Mingkun Jiang, Chen Hua, Chunmao Jiang, Runxin Niu

**Affiliations:** ^1^ Hefei Institutes of Physical Science, Chinese Academy of Sciences, Hefei, China; ^2^ Science Island Branch, University of Science and Technology of China Country, Hefei, China; ^3^ Institute of Computer Science, Chinese Academy of Sciences, Beijing, China

**Keywords:** cherry tomato picking robot, object detection, depth separable convolution, residual module, coordinate attention mechanism, DSP-YOLOv7-CA

## Abstract

The typical occlusion of cherry tomatoes in the natural environment is one of the most critical factors affecting the accurate picking of cherry tomato picking robots. To recognize occluded cherry tomatoes accurately and efficiently using deep convolutional neural networks, a new occluded cherry tomato recognition model DSP-YOLOv7-CA is proposed. Firstly, images of cherry tomatoes with different degrees of occlusion are acquired, four occlusion areas and four occlusion methods are defined, and a cherry tomato dataset (TOSL) is constructed. Then, based on YOLOv7, the convolution module of the original residual edges was replaced with null residual edges, depth-separable convolutional layers were added, and jump connections were added to reuse feature information. Then, a depth-separable convolutional layer is added to the SPPF module with fewer parameters to replace the original SPPCSPC module to solve the problem of loss of small target information by different pooled residual layers. Finally, a coordinate attention mechanism (CA) layer is introduced at the critical position of the enhanced feature extraction network to strengthen the attention to the occluded cherry tomato. The experimental results show that the DSP-YOLOv7-CA model outperforms other target detection models, with an average detection accuracy (mAP) of 98.86%, and the number of model parameters is reduced from 37.62MB to 33.71MB, which is better on the actual detection of cherry tomatoes with less than 95% occlusion. Relatively average results were obtained on detecting cherry tomatoes with a shade level higher than 95%, but such cherry tomatoes were not targeted for picking. The DSP-YOLOv7-CA model can accurately recognize the occluded cherry tomatoes in the natural environment, providing an effective solution for accurately picking cherry tomato picking robots.

## 1 Introduction

Cherry tomatoes, also known as small tomatoes, have high nutritional value. The national cultivation area is approximately 1 million hectares, with an annual production of about 61 million tons, accounting for 35% of the global tomato production. The total output value of cherry tomatoes has reached 10 billion yuan, accounting for 12% of China’s total vegetable output. The average per capita consumption is 21 kilograms per year FAO (2018); Zhang (2014); Feng et al. (2022). However, the manual harvesting cost of cherry tomatoes is about 10,500 yuan per hectare, accounting for over 30% of the total production cost. Timely harvesting of cherry tomatoes is necessary to ensure food quality Zheng et al. (2021). Agricultural harvesting robots provide a new approach to the mechanized harvesting of cherry tomatoes. Unlike other fruits, ripe cherry tomatoes have light red and smooth skin. They grow in complex natural environments, mainly in clusters with many overlapping fruits and obstructions from leaves and branches. The obstruction of cherry tomatoes is one of the most significant factors affecting accurate harvesting by harvesting robots.

More and more studies have utilized deep learning methods to solve the cherry tomato detection problem. Yan et al. (2021) and Feng et al. (2022); Feng et al. (2021) segmented tomatoes for detection based on MsakR-CNN model. Yuan et al. (2020) proposed a robust SSD-based cherry tomato detection algorithm for greenhouse scenarios with an average accuracy of 98.85% but with average detection speed. Wang et al. (2022) proposed an algorithm for tomato ripening detection in complex scenarios based on Faster R-CNN, with an average accuracy of 96.14%. Lv et al. (2023) proposed a cascade deep learning-based tomato flower and fruit detection method with an average recognition rate of 92.30%.The YOLO model has become a research hotspot due to its advantages of fast detection speed. Zhang et al. (2021) based on the YOLOv4 model, the connectivity relationship between the tomato bunches and the corresponding fruit stems to achieve the fast recognition of tomato fruit stems. Zheng et al. (2022) based on the YOLOv4 model using a depth separable convolutional module to improve the backbone network and SPP module, the detection accuracy is 94.44%, but the number of parameters is large. Mbouembe et al. (2023) based on the YOLOv4-tiny model, it utilizes a smaller SPP module to increase the sensory field with an accuracy of 96.35%. He et al. (2022) proposed a tomato detection method based on improved YOLOv5 with an average accuracy of 96.87%. Yang et al. (2022); Zhang et al. (2023b) realized tomato detection by integrating the CBAM attention module into the backbone network part of the YOLO model, which gives more attention to tomato features, but the detection effect is average. Liu et al. (2023) proposed a tomato detection model with a deep convolutional structure to improve the target recognition accuracy while achieving sparsity of model parameters.

In occluded fruit detection, the optimized model can improve the detection accuracy due to the lack of feature information Saedi and Khosravi (2020). Sa et al. (2016) utilized an improved Faster R-CNN model to recognize occluded fruits and obtained a high F1 score; however, the model structure was too complex, and the detection time was long. Yu et al. (2019) successfully detected occluded strawberries with a detection accuracy of 89.5% by migrating the Mask R-CNN model for training. Xu (2013) utilized a Support Vector Machine (SVM) to detect strawberries with 87% accuracy. However, this method can only recognize slightly occluded strawberries. Wang and Long (2023) implemented the detection of occluded citrus using the improved YOLOv3, which could not accurately recognize citrus with a large occluded area. Gai et al. (2023) achieved target detection of occluded cherries based on the YOLOv5s model by adding an RFB module to enhance the shallow feature information. Zhang et al. (2022) effectively improved the detection of occluded small targets by introducing a decoupled head structure on YOLOX. The feature extraction ability of occluded targets can be enhanced by integrating the attention mechanism module in the model Chen et al. (2023). Su et al. (2022) established a tomato ripeness recognition model combining depth-separable convolution and squeeze-excitation attention mechanism module, with a detection accuracy of 97.5%. Khan et al. (2023) designed a convolutional converter-based method for occluded tomato image detection, which performs well in terms of µIoU, µDC, mAP and AUC. Khan et al. (2023) designed a CSPNet structure with hybrid attention based on the YOLOv4-Tiny model fusing CBAM branches in the large residuals of CSPDarknet, which is effective for occluded tomato detection.

Although some studies have been conducted on occluded cherry tomato detection, the current model detection accuracy and efficiency still need to be improved to meet the requirements of cherry tomato detection under picking conditions. It is found that enhancing the SPP network and backbone network to detect occluded small targets based on the YOLO detection model is an effective means, and the use of the attention mechanism module and lightweight convolutional kernel can focus on the critical information, obtaining a better characterization ability and model lightweight. Therefore, this study takes cherry tomato picking robot detection in the natural environment as the theme and occluded cherry tomatoes as the primary research object to improve the detection accuracy of occluded cherry tomatoes in the natural environment. An efficient and stable DSP-YOLOv7-CA detection model is constructed through multi-group controlled experiments, and the DSP-YOLOv7-CA model proposed in this study makes the following five main contributions to existing models:

1. Cherry tomatoes in a natural environment were collected and screened, and various occlusion situations of cherry tomatoes were defined in detail. A new cherry tomato dataset (TOSL) was constructed by offline data augmentation and image labelling operations, and online data augmentation methods further enhanced the diversity of the data.
2. A new cherry tomato recognition model, DSP-YOLOv7-CA, is proposed. The performance of the DSP-YOLOv7-CA model is examined in cherry tomatoes with different occlusion levels.
3. A deep residual DSP-Backbon network with multi-scale detection is designed in the backbone network. The feature information of the original residual layer and the deep separable convolutional layer is utilized for fusion, and the feature information is reused through the DSP-Multiblock module to accelerate the convergence speed of the model.
4. In the spatial pyramid network, the DSP-SPPF module with fewer parameters and better performance is designed, and the DSP-SPPF module solves the problem of the loss of small target information in different pooled residual layers, improves the generalization ability of the model and reduces the number of parameters of the network.
5. A coordinate attention mechanism (CA) layer is introduced at critical positions in the enhanced feature extraction network to better extract features of complex small targets and improve the detection accuracy and speed of the network.

## 2 Materials and methods

### 2.1 Image collection

#### 2.1.1 Image acquisition methods

**Figure 1:**
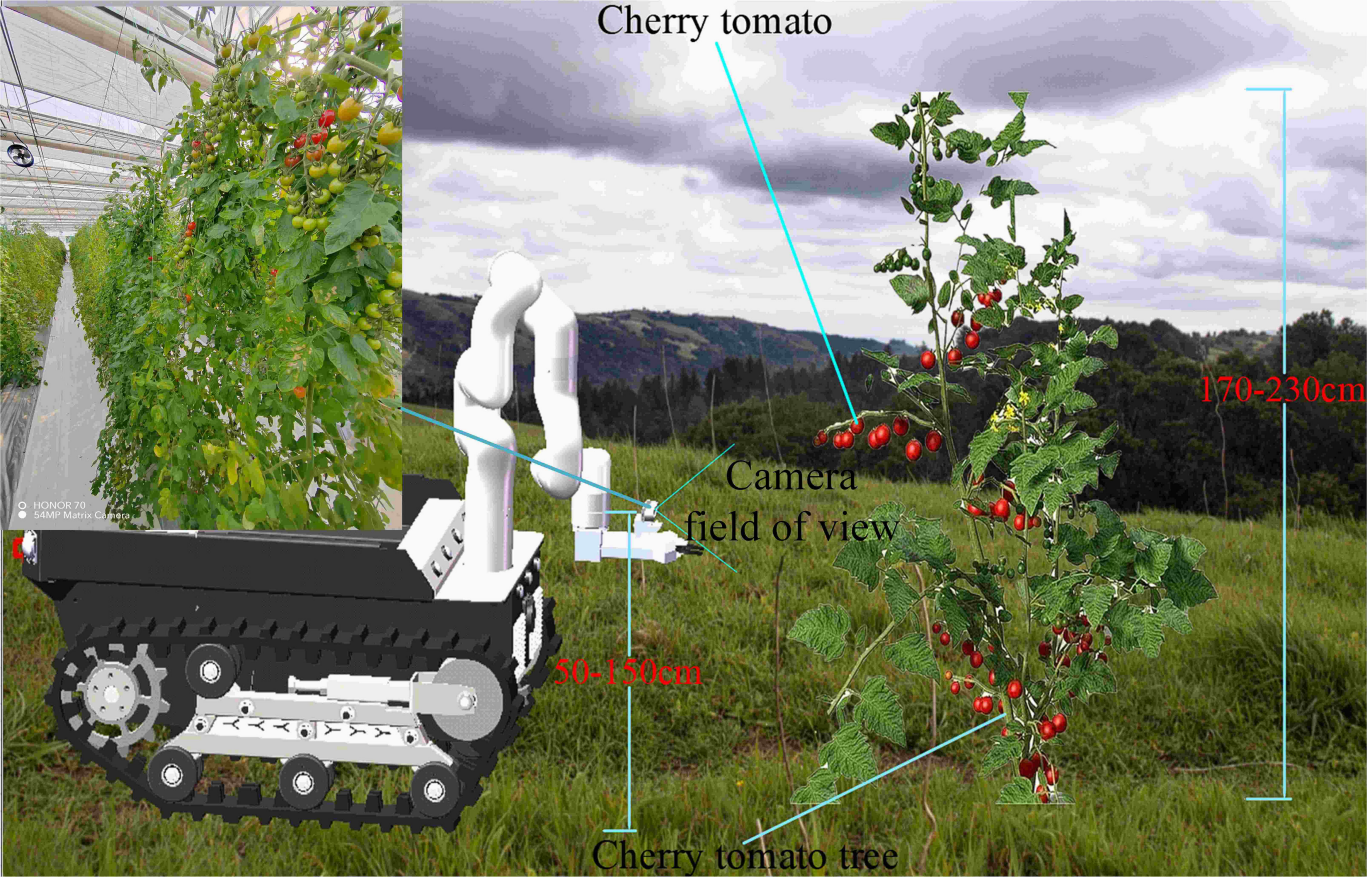
Camera field of view of a cherry tomato robot at work.

In this study, the variety of cherry tomatoes from Shandong Province was selected as the research object, and the variety of cherry tomatoes was selected as Pink Pei Pei. During January and March 2023, several cherry tomatoes with good growth, ripe and intact fruits were selected for photographing according to the field-of-view angle of the camera during the working process of the cherry tomato picking robot at the National Saline and Alkaline Land Facility Agricultural Testing Experimental Base, as shown in **Figures 1**. The shooting was done with an ultra-wide-angle lens equipped by a 54 MP matrix camera of Honor 70. The data in the figure contains images of cherry tomatoes with different fruit densities and at different periods. We finally captured 1293 high-resolution images of ripe cherry tomatoes in JPG format with a resolution of 3072 pixels × 4096 pixels.

**Figure 2:**
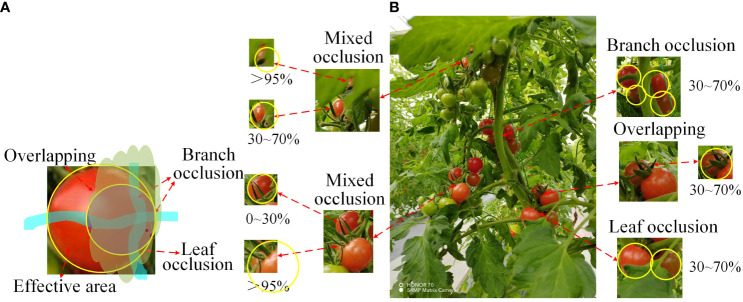
The way cherry tomatoes are shaded. **(A)**: Analogue masking method. **(B)**: Actual masking method.

The main objective of this study is to improve the recognition accuracy of occluded cherry tomatoes in natural environments. This paper pays special attention to the branch-obscured ripe cherry tomato images when selecting cherry tomato images. For the positional relationship between fruits and obstacles in cherry tomato picking robots, this paper defines four types of occlusion areas and four types of occlusion modes, which are: 0-30%, 30-70%, 70-95%, 95-100%, branch-obscured, leaf-obscured, fruit overlapped, and mixed-obscured Yang et al. (2019). As shown in **Figure 2**, cherry tomato branches will pass through the middle or edge of the fruit, fruit overlap will overlap a portion of the tomato features, and be shaded by foliage irregularly and, more commonly, with varying sizes of shaded areas. The yellow circle markers are the size of the complete cherry tomato silhouette, and the occlusion area was determined based on the ratio of the size of the surface features that the camera could not detect to the size of the complete silhouette. In the end, this paper retains 581 clear images of occluded cherry tomatoes, which enables the YOLO target detection algorithm to comprehensively learn the surface features of cherry tomatoes under various occlusion methods.

#### 2.1.2 Data augmentation

Model training requires a large amount of data, and various offline data augmentation methods are commonly used to expand the dataset Zhang et al. (2023a). However, the data diversity of these conventional methods is generally insufficient. Therefore, in this paper, we use offline and online augmentation to augment data from shaded cherry tomatoes in natural environments, fusing multi-scale features to increase data diversity.

**Figure 3:**
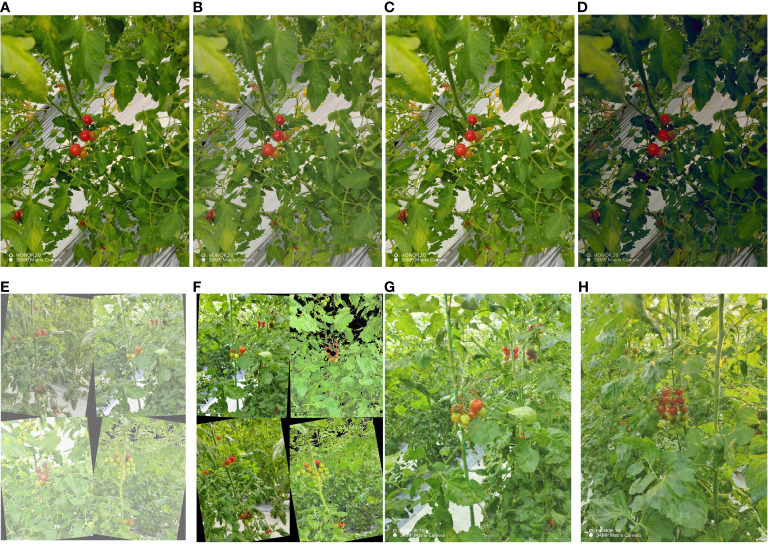
Cherry tomato images. **(A–D)** Offline augmentation images with changes in lighting and added noise. **(E, F)** Online mosaic augmentation images. **(G, H)** Online mixup augmentation images.

**Figure 4:**
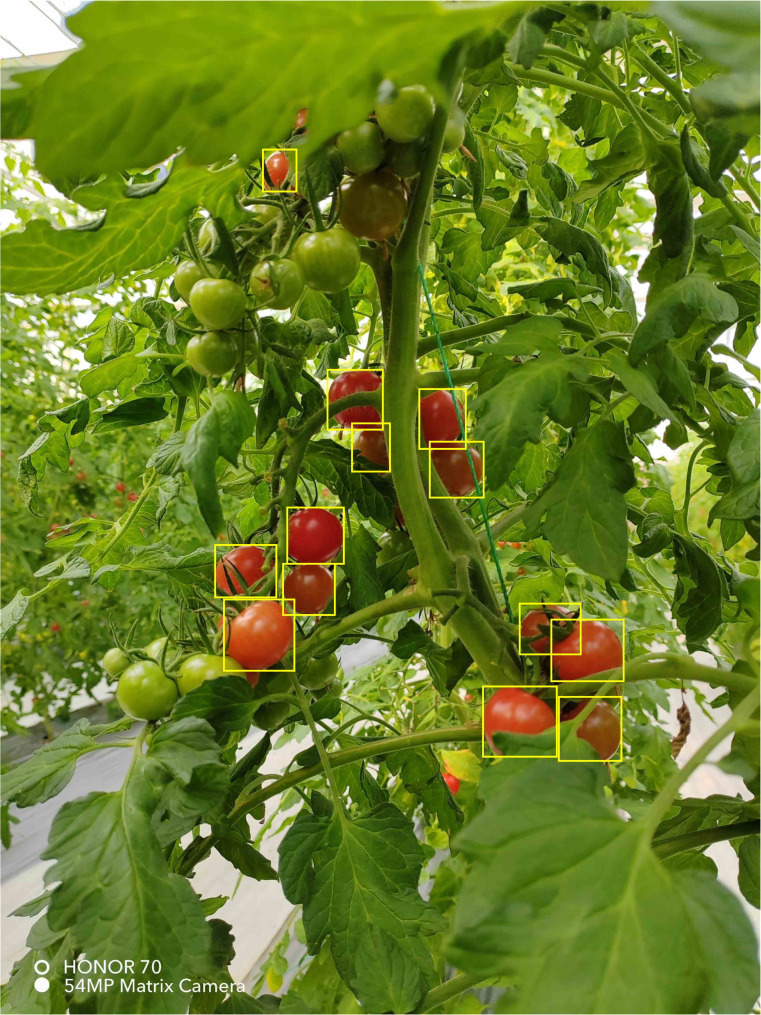
Labeled images.

Offline augmentation is a data enhancement method performed before model training, which includes the following two methods: (1) Light change: change the saturation and brightness of the image to simulate the brightness difference between different weather in the daytime environment. (2) Adding noise: adding Gaussian noise to the image data to simulate the noise during the shooting process and reduce the high-frequency features to prevent the overfitting phenomenon. Through the above two methods, the offline data were expanded to 2334 images, and the expanded images are shown in **Figures 3A–D**. LabelImg software labelled the ripe cherry tomatoes with less than 95% of the occluded area in these images and obtained 14623 labelled instances. Since it is also more difficult for the human eye to discriminate cherry tomatoes with 95 100% of the occluded area, they are not used as the detection target of this model. The labelled areas are the smallest rectangles around the cherry tomatoes. The labelled images are shown in **Figure 4**. The dataset of occluded cherry tomatoes in the natural environment (TOSL) was constructed by offline amplification and image labelling work.

A combination of Mosaic and Mixup image augmentation methods was used in online augmentation Zhang et al. (2023a). Mosaic image augmentation enriches the background of the detected object by stitching four images together, including colour gamut changes, rotations, adding noise, and killing features. Mixup image augmentation creates new training samples by mixing different images to improve the model’s generalization. Too large an amplification probability can over-process the image data, resulting in the loss of feature information for cherry tomatoes with large occluded areas. Too small amplification probabilities reduce the number of amplifications and do not fully utilize the enhancement they provide. Therefore, a 50% probability was set for Mosaic and Mixup data amplification during each iteration, as shown in **Figures 3E–H**.

### 2.2 Model building

#### 2.2.1 YOLOv7 model

**Figure 5:**
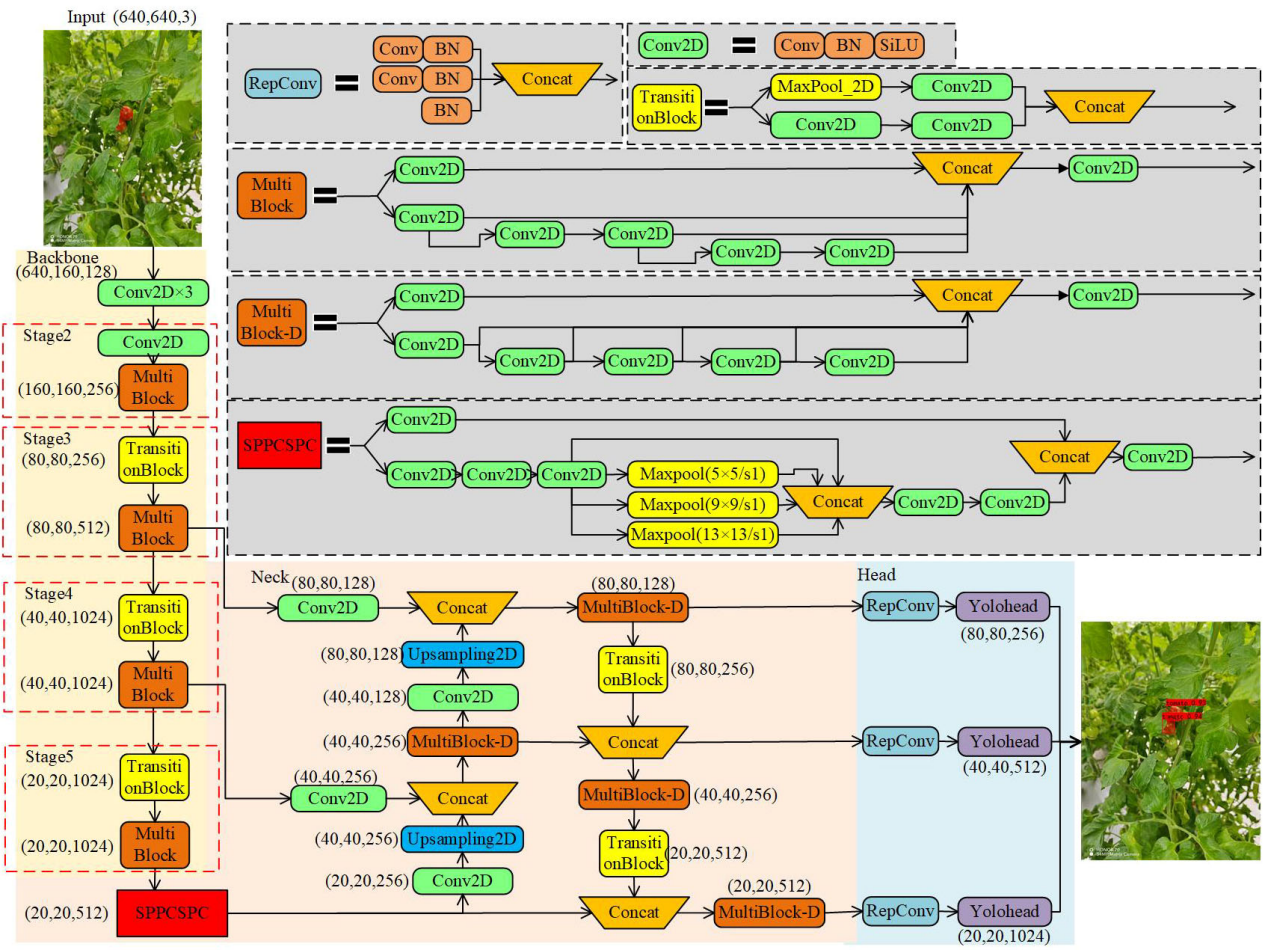
YOLOv7 structure diagram.

The YOLOv7 model has higher detection accuracy and detection speed Wang et al. (2023). In **Figure 5**, the YOLOv7 model is demonstrated with a multi-branch stacking structure for feature extraction in the backbone network and the enhanced feature network, and the model has a denser jump connection structure. The backbone consists of several Conv2D modules, TransitionBlock modules and MultiBlock modules, where the Conv2D module consists of Conv+BN+SiLU. The innovative downsampling structure TransitionBlock module (composed of Maxpool and Conv2D) is used to extract and compress feature maps simultaneously. MultiBlock module employs a residue-like stacking structure composed of multiple Conv2Ds, capable of extracting features at different scales. The enhanced feature extraction network uses a PAFPN structure, similar to YOLOv5, except that the CSP module replaces the MultiBlock-D module. Both MultiBlock-D and the MultiBlock in the backbone have similar structural composition, with only a difference in the number of Concatenations. The model outputs three different sizes of prediction results to realize multi-scale prediction for multiple targets.

#### 2.2.2 Depthwise separable convolution

**Figure 6:**
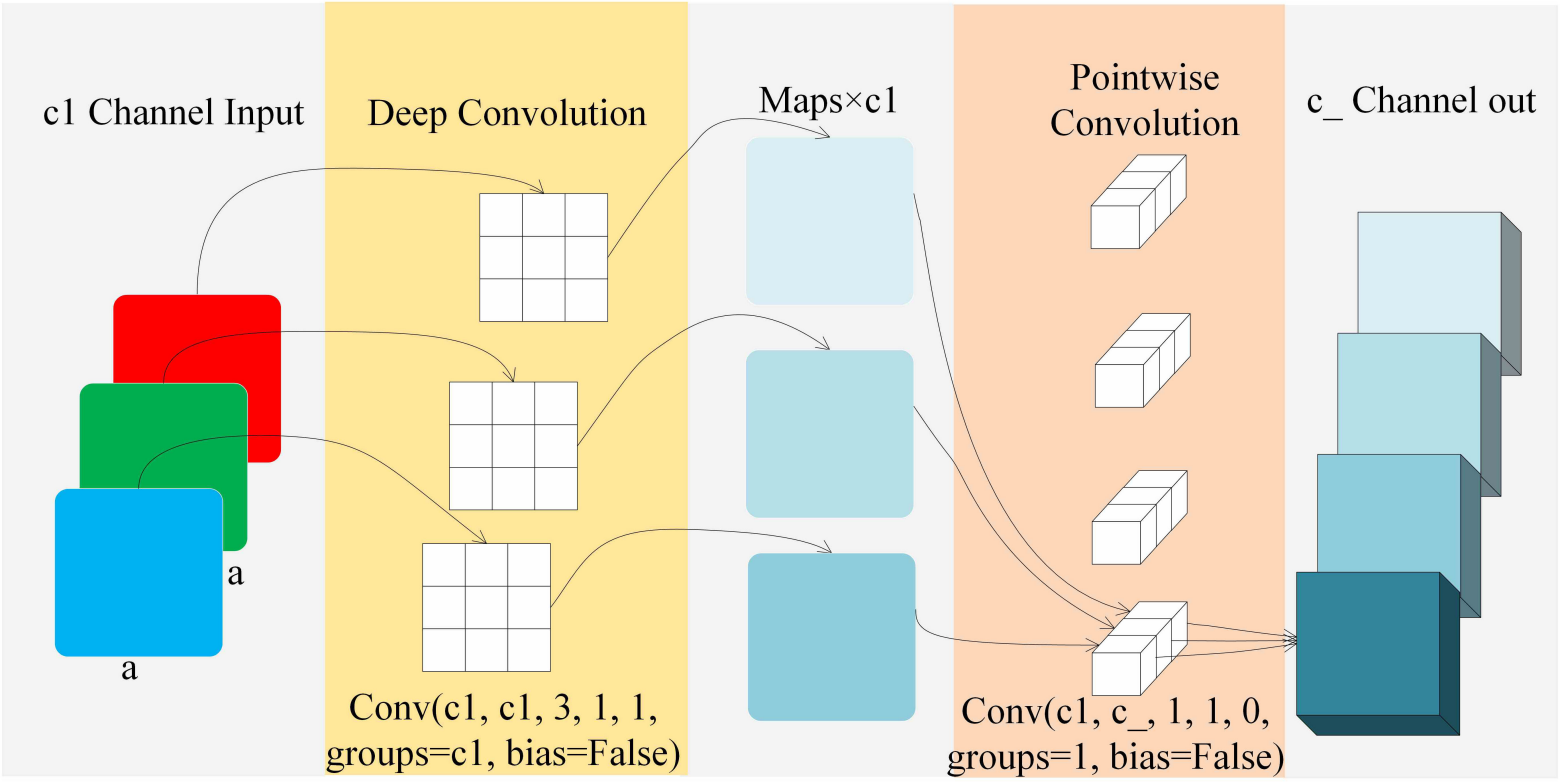
Depthwise separable convolution.


In 2018, Sandler et al. (2018) proposed depthwise separable convolution. As shown in **Figure 6**, depthwise separable convolution consists of depthwise and pointwise convolution. In the depthwise convolution layer, assuming the input feature has dimensions of a × a × c1, where c1 is the number of channels, the convolutional kernel in this paper has parameters of 3 * 3 * 1 * c1. The output feature after depthwise convolution has dimensions of a × a × c1. During the convolution, each channel corresponds to only one convolutional kernel, so:

(1) FLOPs1=c1×a×a×3×3

In the pointwise convolution, the input is the feature after depthwise convolution, with dimensions of c1 × a × a. The convolutional kernel parameters are c2 × 1 × 1 × c1. The output dimensions are c1 × a × a. During the convolution process, a standard 1 × 1 convolution is applied to each feature, so:


(2) $$FLOPs2=c_{-}\times a\times a\times c1$$

Therefore, the ratio of the parameter quantity of depthwise separable convolution to the parameter quantity of standard convolution is shown in Formula 3.


(3) $$\frac{c1\times a\times a\times 3\times 3+c_{-}\times a\times a\times c1}{a\times a\times 3\times 3\times c\times c1}=\frac{1}{c_{-}}+\frac{1}{9}$$

Compared to standard convolution, depthwise separable convolution has the characteristics of parameter sharing and sparse interactions. It can share the trained convolutional kernel weights, reducing the number of parameters. At the same time, it connects partial inputs to better capture local features of the input, enabling the learning of spatial and channel features, and providing better representational power.

#### 2.2.3 Residual neural network

In 2016, He et al. (2016) proposed residual networks that can improve the model’s accuracy by increasing the network’s depth. The basic principle of residual networks is to construct an identity mapping using residual units, i.e., y = F(x) + x, where F(x) represents the nonlinear transformation part of the network, which adds the input signals to the output through direct connections across the layers, and x is the original transmission part, which retains the original feature information and achieves a better feature fusion. This structure ensures that parameter updates do not suffer from the problem of disappearing gradients or gradient explosions.

#### 2.2.4 CANet

When performing feature map fusion, obtaining high-level semantic features and low-level contour features is beneficial. For the detection of occluded cherry tomatoes, it becomes difficult to enhance the feature extraction network to obtain features from the global features, which reduces the detection accuracy of the model. Therefore, the spatial attention mechanism (CBAM) significantly improves the model performance Li et al. (2020). However, using CBAM instead of the fully connected layer for feature map encoding usually ignores the location information, essential for generating spatially selective attention maps. The Coordinate Attention Mechanism (CA) Hou et al. (2021) decomposes channel attention into two one-dimensional feature encoding processes that aggregate features in two spatial directions, respectively. Then, it encodes the generated feature maps into a pair of direction-aware and location-sensitive attention maps that can be applied complementarily to each other.

### 2.3 Model improvement

YOLOv7 can solve the problem of uneven volume size of cherry tomato images collected by picking robots. The backbone network adopts a more extensive network structure, which may lead to problems such as missed detection and false detection due to the complex occlusion relationship between cherry tomatoes and branch foliage; the spatial pyramid network uses maximum pooling layers parallel to each other, which pays more attention to detecting large targets and is not sensitive enough to detect small targets. The enhancement of the feature extraction network is too deep, and it can not gather small target features in the processing of extracting small features and carrying out zoom-in and zoom-out, resulting in a decrease in detection accuracy and speed. Since this study aims to solve the problem of occluded cherry tomato detection, specific occluded small targets must be considered, and optimizing the original network structure is more important. The optimization method based on the YOLOv7 model in this study includes the following points:

1) In the backbone network, a deep residual DSP-Multiblock module with multiscale detection replaces the MCB module in the last two layers of the backbone network. In the DSP-Multiblock module design, the null residual edge replaces the convolution module on the Multiblock module, and a new depth-separable convolution module is added to realize the fusion of the feature information of the original residual layer and the depth-separable convolution layer further to improve the representation capability of the occluded cherry tomato and to accelerate the convergence speed of the model.
2) The DSP-SPPF module with fewer parameters but superior performance is used in the spatial pyramid network. After comparing the two latest SPP modules, a new depth separable convolution module is added to the better-performing SPPF module and replaces the SPPCSPC module of the original model, which solves the problem of the loss of small target information by different pooled residual layers, improves the generalization ability of the model, realizes the reuse of feature information, and reduces the number of parameters of the network.
3) A coordinate attention mechanism (CA) layer is introduced at critical positions in the enhanced feature extraction network. The coordinate attention mechanism can improve the attention to overlapping and occluded targets and better extract the features of complex small targets, thus improving the detection accuracy and speed of the network.

#### 2.3.1 DSP-backbone network

**Figure 7:**
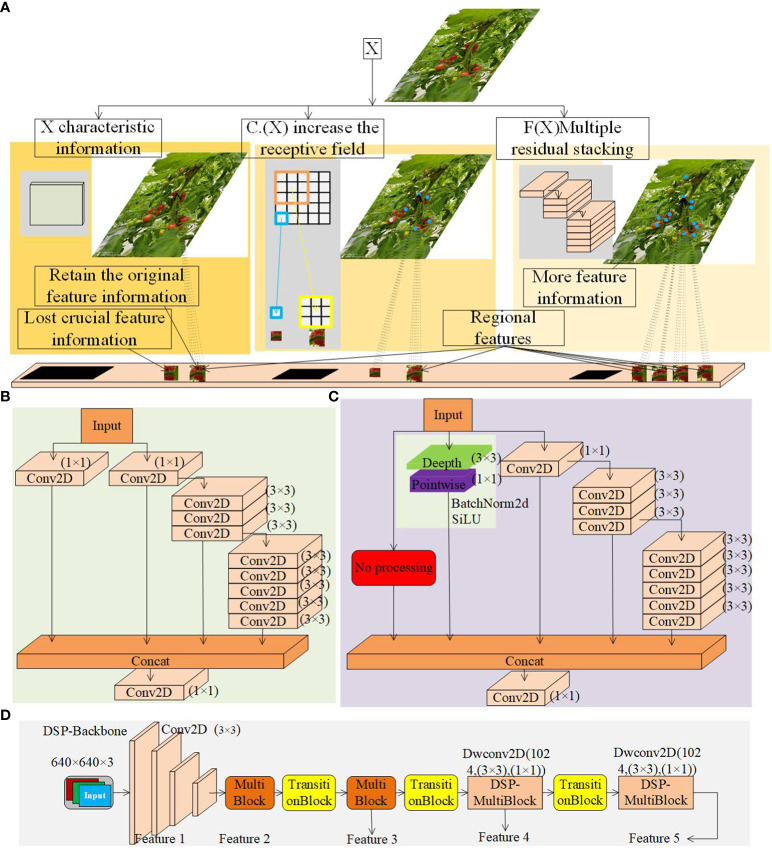
**(A)** DSP-MultiBlock Module Convolution Principle. **(B)** Original MultiBlock Module. **(C)** DSP-MultiBlock Module. **(D)** DSP-Backbone Networks.

In the backbone network, the MultiBlock module realizes the depth increase of the model through the residual-like network, which consists of multiple Conv2D modules, as shown in **Figure 7B**, and the structure of the residual-like network is y = F(x) + C(x), which is prone to lose the critical feature information compared to the residual network y = F(x) + x. Therefore, in this paper, we first utilize the empty residual edges to replace the original residual edges on the MultiBlock module with the Conv2D module, which uses the jump connection of the residual network, which can bypass the occluded region to directly transmit the unoccluded feature information and retain the original feature information. The backbone network gradually completes the 32-fold downsampling operation through four MultiBlock modules. However, if the network layer is too deep, it reduces the possibility of complete information retention, thus weakening the feature extraction capability for small and partially occluded targets. To improve the feature extraction ability, in this paper, we add a branch of depth-separable convolution module to the MultiBlock module with a depth convolution kernel size of (3,3) and a point-by-point convolution kernel size of (1,1), which increases the sensory field of the occluded cherry tomatoes. We name the modified module the DwConv2D module, which consists of the Conv1 (depth-separable convolution), the BN (batch normalization) and SiLU. As shown in **Figures 7A, C**, the new network structure is y = F(x) + x + C.(x), which ensures mutual fusion in the same dimension, and in this paper, we name the modified module DSP-MultiBlock.

The DSP-MultiBlock module adopts a deep residual structure. The feature maps are small in the last two layers of the MultiBlock module in the backbone network, which leads to a relatively sizeable sensory field. The recognition effect is better for large targets, but the recognition effect is average for minor marks. In addition, after multiple downsampling, the detailed information on the high-level features is seriously lost. Therefore, replacing the MultiBlock module of stage 4 and stage 5 in the backbone network with the DSP-MultiBlock module can aggregate the detail information features and realize the conversion between detail and semantic information, avoiding the deeper class residual network, which reduces the detail feature extraction ability. The improved backbone extraction network is called DSP-Backbone, as shown in **Figure 7D**.

#### 2.3.2 DSP-SPPF spatial pyramid network

**Figure 8:**
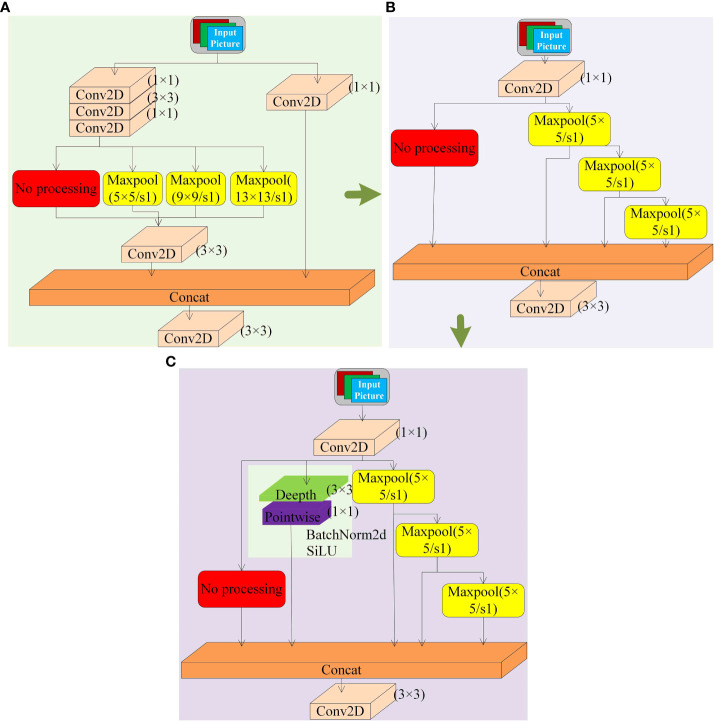
Several SPP modules. **(A)** SPPCSPC Module. **(B)** SPPF Module. **(C)** DSP-SPPF Module.

As shown in **Figure 8A**, the SPPCSPC module of the YOLOv7 model acquires feature information through parallel convolution and maximum pooling kernels parallel to each other. This similar convolution structure can capture the local network and patterns of the input data, especially for the strong representation of features such as edges and textures in the occluded cherry tomato image. However, such a modular structure is too complex and can easily lead to the loss of more minor feature information. For example, since the size of the occluded area of cherry tomatoes varies, the essential information provided is also different. When the occlusion area is large, the target feature information is small. After the feature extraction is completed in the backbone network, some of the feature information may have been lost. When the maximum pooling operation is performed in the SPPCSPC module in parallel with each other, almost all of the feature information is lost, which increases the probability of partial occlusion targets being missed. Compared with this structure, the SPPF module of YOLOv8 adopts a top-down maximum pooling kernel stacking structure, which introduces jump connections and reduces multiple layers of convolutional modules, significantly reducing the number of references and improving the detection speed, as shown in **Figure 8B**. However, the parallel convolutional structure is missing, which increases the probability that partially occluded targets are missed. To compensate for the loss of accuracy due to the loss of convolutional branches, this paper adds a depth-separable convolutional module, DwConv2D, as a parallel convolutional branch on top of the SPPF module. As shown in **Figure 8C**, by increasing the sensory field layer by layer, it captures a broader range of contextual information, which helps to extract more advanced semantic features, enabling the model to understand more complex image contents, further improving the detection accuracy of occluded tomatoes, while reducing the model parameters. The modified module is named the DSP-SPPF module.

#### 2.3.3 Introduction of the enhanced feature extraction network with CANet

**Figure 9:**
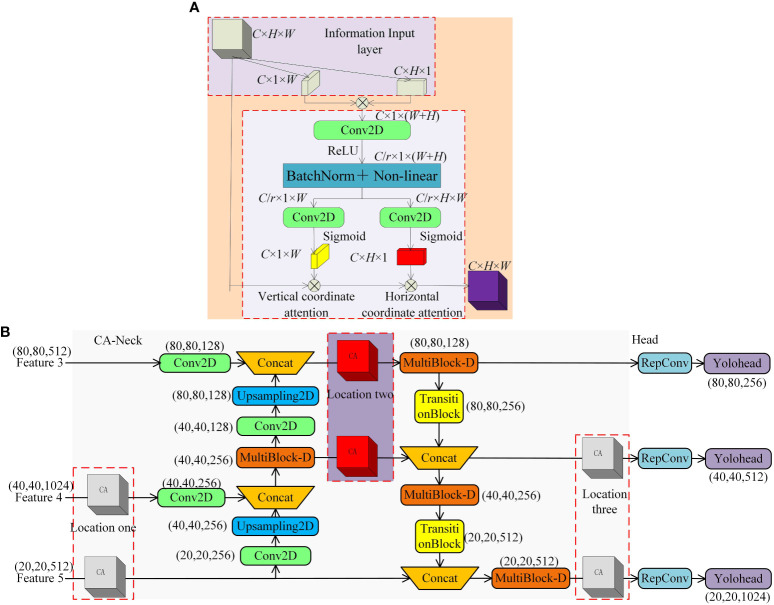
CANeck. **(A)** CA Module. **(B)** Enhanced Feature Fusion Network with Coordinate Attention Mechanism.

To increase the model’s sensitivity to occluded cherry tomato features, this paper employs a coordinate attention mechanism that combines positional information with channel information and is applied to enhance the critical position of the feature extraction network. The method increases the attention on overlapping and occluded targets that are difficult to recognize by assigning higher weights. As shown in **Figure 9A**, the coordinate attention mechanism consists of information embedding and attention generation. In the information embedding stage, all channels of the input feature map are average pooled along the horizontal and vertical coordinate directions, respectively, and feature maps with dimensions C × H × 1 and C × 1 × W are acquired. In the attention generation stage, the two developed feature maps are spliced into a C × 1 × (H + W) feature map. Then, their channel dimensions are compressed from C to C/r dimensions with shrinkage r using 1 × 1 convolution and nonlinear activation using the ReLU function. Next, the acquired results were decomposed along the spatial dimension into a C/r × H × 1 horizontal attention tensor and a C/r × 1 × W vertical attention tensor. The channel dimension is then raised from the C/r dimension to the C dimension using two sets of 1 × 1 convolutions, and the Sigmoid function is used for nonlinear activation. Finally, the two acquired attention maps, C × H × 1 and C × 1 × W, are multiplied with the input feature maps to complete the imposition of coordinate attention.

Considering the complexity of the natural environment, this paper introduces the coordinate attention mechanism to strengthen the feature extraction network to fully acquire feature information at different scales at the information intersection at position 2. As shown in **Figure 9B**, under the imposition of two other directional attention maps, it can determine whether the target exists in the corresponding rows and columns, which in turn improves the network’s recognition effect for dense targets and, at the same time, mitigates the degradation of the detection accuracy caused by the occlusion of branches and leaves. Notably, the CA modules at positions 1 and 3 of the network only apply to the subsequent illustration of the control experiments and do not serve as part of the final network structure.

### 2.4 Model training

#### 2.4.1 Training method and platform

In this experiment, the PyTorch deep learning framework is built on a hardware platform equipped with Intel 13th Core(TM) i5-13600KF and NVIDIA GeForce RTX 3090 (with 24GB video memory) and running on the Windows 10 operating system. The target detection model for occluded cherry tomatoes was implemented using related libraries such as CUDA 12.1 and OpenCV, and the model was trained and tested.

#### 2.4.2 Training strategy

**Figure 10:**
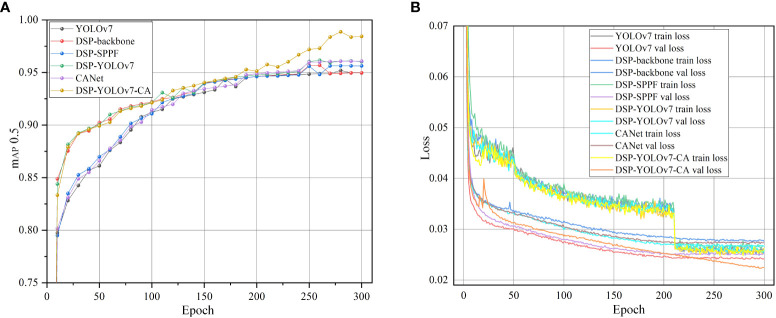
Training process. **(A)** Precision curve. **(B)** Loss curve.

In this study, the dataset is divided into training, validation, and test set in an 8:1:1 ratio, and then the images are inputted into the feature space with the size of 640×640. In this study, the pre-training weights of YOLOv7 are used for training, and the training data are saved in the model weights file; the first 50 iterations of the model are frozen for training, the batch size is set to 8 in the freezing phase, and the model is thawed for training for 250 times, the batch size is set to 6, and the model is trained for a total of 300 times. Perform a validation every 10 iterations and record the relevant information. At the end of training, the weights file of the target detection training model is saved, and the model’s performance is evaluated on the test set. The label translation rate is 0.005, the maximum learning rate of the model is 0.01, the minimum learning rate is 0.0001, the gradient descent parameter is 0.937 using the SGD optimizer, the weight decay rate is set to 0.0005, and the type of learning rate decay is cosine decay. **Figure 10A** shows the average accuracy curve during the training process of the primary model; after 200 iterations, the average accuracy of DSP-YOLOv7-CA is significantly higher than the other models, and it reaches the maximum value in the 280th iteration, and **Figure 10B** shows the loss rate curve during the training process of the primary model, in the first 10 Epochs, the model is converging rapidly, and in the past 210 Epochs, The loss function is stable. The difference between the two accuracies is close to 0, indicating that the model has reached the fitting state and achieved good training results.

#### 2.4.3 Experimental evaluation indicators

In testing the effectiveness of the model, Precision, Recall, F1, mAP, params, FLOPs, and FPS are used in this paper to evaluate the recognition performance of occluded cherry tomatoes. Precision is the probability of actual positive samples among the samples predicted to be positive by all the predictions. Recall is the probability of being heralded as a positive sample among the actually positive pieces. Where *T_P_* refers to the number of positive models correctly predicted, *F_P_* refers to the number of negative samples incorrectly expected as positive samples. *F_N_* refers to the number of positive samples that are expected as negative samples. The formula for its calculation is:


(4) $$P=\frac{T_{P}}{T_{P}+T_{P}}$$

(5) $$R=\frac{T_{P}}{T_{P}+F_{N}}$$

The calculation of The F1 score is related to the values of precision and recall, and the level of The F1 score represents the stability of the model, which is calculated by the formula:


(6) $$F1=\frac{2PR}{P+R}$$

The mAP is the average of the mean accuracy and AP of each category, which is calculated by the formula:


(7) $$m_{AP}=\frac{1}{m}\int_{0}^{1}P(R)dR$$

FPS refers to the number of frames transmitted per second, and avg is the total inference time; the number of inferences in this study is 100 and FPS is obtained by the reciprocal of avg, which is calculated as follows:


(8) $$FPS=\frac{1}{avɡ}$$

Parameters are another critical measure of model complexity. A higher number of parameters in a model means that the model requires more computational resources and data for training and inference. For example, more GPU memory is needed to train the model, and each number corresponding to the weight matrix inside the convolution and full join used in the model is a component of the number of parameters.

*C*_0_ denotes the number of output channels, *C_i_* denotes the number of input channels, *k_w_* denotes the convolution kernel width, and *k_h_* denotes the convolution kernel height, which is computed by the formula:


(9) $$params=C_{0}\times (k_{w}\times k_{h}\times C_{i}+1)$$

The number of floating-point operations (FLOPs) is the amount of model computation, which refers to the number of floating-point functions required to run a network model once. FLOPs are usually used to measure a model’s computational efficiency and speed. For example, when deploying a model on a tomato-picking robot, the device’s limitations need to be considered. If the model’s computation is too large, it will lead to a long inference time, which is unsuitable for practical applications. W and H denote the length and width of the feature map, respectively. Its calculation formula is:


(10) $$FLOP_{s}=\left[\left(C_{i}\times k_{w}\times k_{h}\right)+\left(C_{i}\times k_{w}\times k_{h}-1\right)+1\right]\times C_{0}\times W\times H$$

## 3 Results

To validate the effectiveness of the method designed in this paper for the cherry tomato detection task, we compared the effects on model performance before and after the imposition of different improvement methods on the cherry tomato dataset (TOSL) in a multi-group controlled experiment.

### 3.1 Comparative experiments with different DSP networks

#### 3.1.1 Comparison of results for different backbone networks

**Figure 11:**
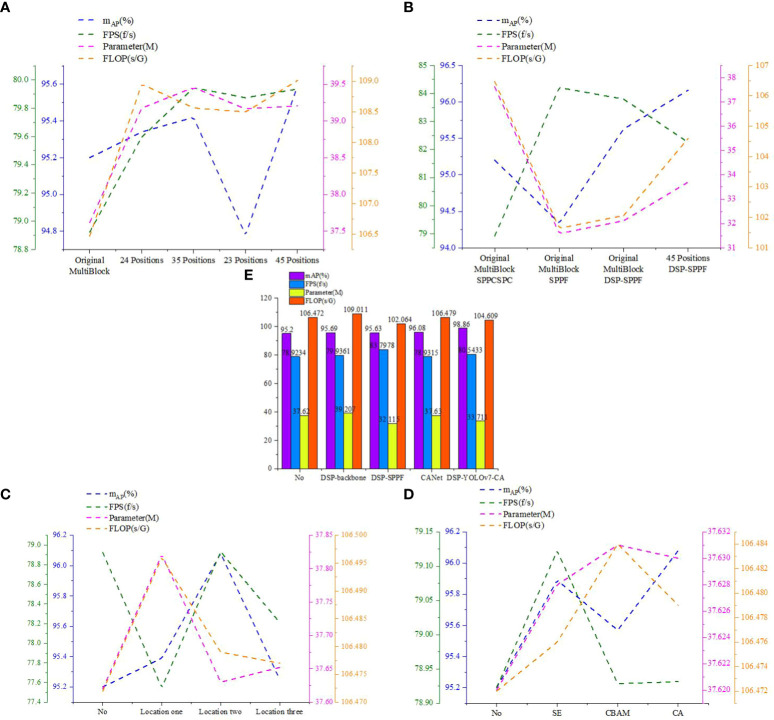
Comparison of the performance of the best model in each method. **(A)** Comparison of different backbone networks. **(B)** Comparison of different SPPs. **(C)** Comparison of applying CA attention mechanisms to different locations. **(D)** Comparison of applying different attention mechanisms to location two. **(E)** Comparison of each best model.

In this section, comparison experiments of DSP-MultiBlock modules at each position are conducted, as shown in **Figure 7D**, where models with different Backbone and other conditions being the same are designed, and the MultiBlock modules at two or two of the two, three, four, and five positions in the original YOLOv7 backbone extraction network are replaced by DSP-MultiBlock modules, respectively. The post-experiment results are shown in **Table 1** and **Figure 11A**. The highest mAP value of 95.69% is obtained by the model when the DSP-MultiBlock module is located in the four-five position, which improves 0.49 percentage points compared to the original model, and the FPS improves by 1f/s. Although the number of parameters has increased by 2.2M compared to the original model, this substitution allows the model to better deal with the balance of the detail information and the semantic information balance, which improves the accuracy on occluded small targets.

**Table 1:** Comparison of detection capabilities of different backbone networks.

Backbone	*m_AP__*0.5(%)	FPS(f/s)	Parameter(M)	FLOPs/G
Original MultiBlock	95.20	78.9234	37.620	106.472
24 instances of DSP-MultiBlock	95.34	79.5952	39.174	108.952
35 instances of DSP-MultiBlock	95.42	79.9456	39.456	108.564
23 instances of DSP-MultiBlock	94.78	79.8744	39.165	108.497
45 instances of DSP-MultiBlock	**95.69**	79.9361	39.207	109.011

The bold font highlights the advantages in 45 instances of DSP-MultiBlock mAP.

#### 3.1.2 Comparison of results for different SPP spatial pyramid networks

The previous section determined that the DSP-MultiBlock module is more effective when it is located at four or five positions. In this section, the comparison experiments of different SPP spatial pyramid networks are conducted by designing Backbone and neck different models with the same conditions of other conditions and replacing the SPPCSPC module of the original YOLOv7 with the SPPF module and the DSP-SPPF module, respectively. The post-experimental results are shown in **Table 2** and **Figure 11B**. The SPPF module reduces the number of parameters by 6M compared to the SPPCSPC and achieves an FPS of 84.2119f/s, with a reduction of 5s/G for floating-point computation. The detection speed is lower than that of the SPPF module when using the DSP-SPPF module, but the mAP value improves by 1.3 percentage points. The detection speed is lower than that of the SPPF module when using the DSP-SPPF module in both the Backbone and spatial pyramid networks. At the same time, the DSP structure improves the average accuracy of the model by nearly one percentage point over the original network, and the FPS improves by 3.3 f/s. In addition, the amount of parameters is reduced by 4M, and the amount of floating-point computation is reduced by 1.8s/G. In summary, the DSP structure formed by adding a stack of pooling layers of depth-separable convolutions and residuals can capture a more extensive range of contextual information; due to the use of residual structure in the SPPF module, a large number of convolutional modules are reduced, the model is more lightweight, and these improved methods achieve significant improvements in terms of average accuracy, FPS, number of parameters, and floating-point computation.

**Table 2:** Comparison of detection capabilities of different SPP spatial pyramid networks.

Backbone	Neck	*m_AP__*0.5(%)	FPS(f/s)	Parameter(M)	FLOPs/G
Original MultiBlock	SPPCSPC	95.20	78.9234	37.620	106.472
Original MultiBlock	SPPF	94.34	**84.2119**	**31.586**	**101.641**
Original MultiBlock	DSP-SPPF	95.42	79.9456	39.456	108.564
45 instances of DSP-MultiBlock	DSP-SPPF	**96.16**	82.2568	33.701	104.602

The bold font denotes which model performs best on a particular metric.

### 3.2 Comparative experiments with different attentional mechanisms

#### 3.2.1 Comparison of results for different positions imposed by the coordinate attention mechanism

For the complexity of the cherry tomato growing environment and the need to further improve the detection accuracy, this section applies the coordinate attention mechanism CA to different positions in the feature fusion network based on the original YOLOv7, as shown in **Figure 9**. By comparing the effects of applying the attention mechanism to varying situations on the detection performance of the model, the results are shown in **Table 3** and **Figure 11C**. By using the attention mechanism at position 2, 0.88 percentage points improve the average accuracy of the model, and the FPS is not reduced but slightly improved. Applying the attention mechanism at position 1 and position 3 improved the accuracy by 0.19 and 0.05 percentage points, respectively. Since position 2 is at the intersection of different scales of information in the enhanced feature extraction network, richer feature information can be obtained compared to position 1 and position 3, thus improving the detection effect of the model.

**Table 3:** Comparison of detection capabilities by applying attention mechanism to different positions.

Apply position	*m_AP_* (%)	FPS(f/s)	Parameter(M)	FLOPs/G
NO	95.20	78.9234	**37.620**	**106.472**
Location one	95.39	77.5485	37.821	106.496
Location two	**96.08**	**78.9315**	37.630	106.479
Location three	95.25	78.2112	37.652	106.477

The bold font denotes which model performs best on a particular metric.

#### 3.2.2 Comparison of results for different attentional mechanisms at position 2

To further validate the performance of different attention mechanisms in position 2, this section conducts a comparison experiment based on the original YOLOv7. The investigation results are shown in **Table 4** and **Figure 11D**, and it can be found that the highest mAP value is achieved when using the CA attention mechanism module. The CA module introduced in this paper uses two one-dimensional attention maps for feature encoding, and embeds feature information at different scales. The model’s sensitivity to dense targets can be effectively improved through this approximation of coordinates, which in turn improves the negative impact of various occlusion situations on detection accuracy in the cherry tomato detection task.

**Table 4:** Comparison of detection capabilities with different attention mechanisms applied.

Attention mechanisms	*m_AP__*0.5(%)	FPS(f/s)	Parameter(M)	FLOPs/G
SE	95.89	**79.1223**	**37.628**	**106.476**
CBAM	95.57	78.9286	37.631	106.484
CA	**96.08**	78.9315	37.630	106.479

The bold font denotes which model performs best on a particular metric.

### 3.3 Ablation experiment

**Figure 12:**
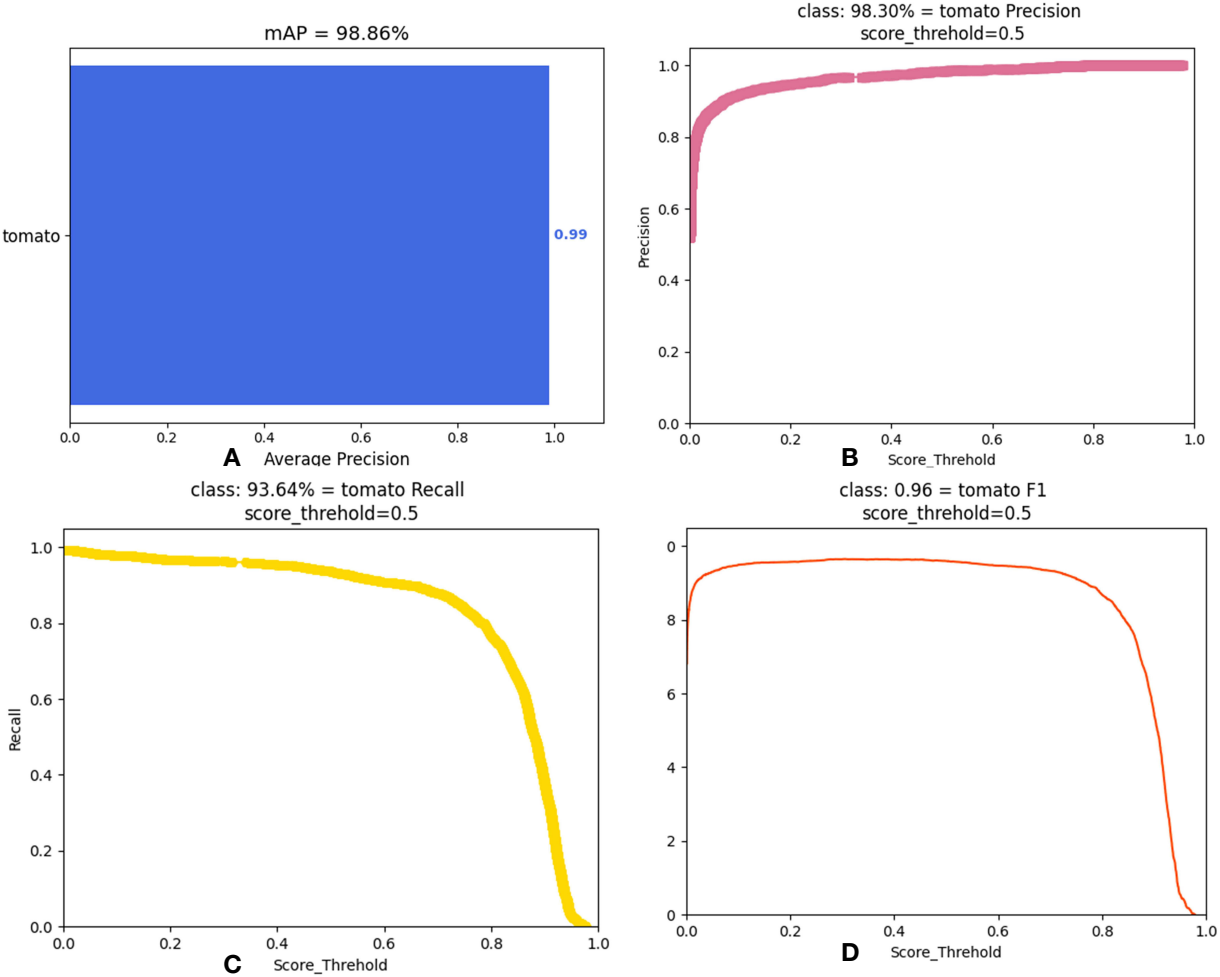
Performance metrics of DSP-YOLOv7-CA. **(A)** mAP value. **(B)** precision rate. **(C)** recall rate. **(D)** F1 value.

This section conducts the comparison experiments of each optimal method and the combination of each optimal strategy. The DSP-YOLOv7-CA model is the optimal model obtained by combining each optimal approach. The results of the experiments are shown in **Table 5**; compared with the original YOLOv7 model, the number of parameters of the DSP-YOLOv7-CA model is reduced from 37.62MB to 33.711MB, and the running speed is reduced from 106.472Gflops to 104.609Gflops, and FPS increased from 78.9234 to 80.5433. As shown in **Figure 11E**, the DSP-YOLOv7-CA is higher than the other best models’ average accuracy, and the mAP value reaches 98.86%. **Figure 12** depicts the obtained performance graphs, including the accuracy P, the recall R, the AP value, and F1 and mAP values. However, DSP-YOLOv7-CA is not the best regarding FPS, Parameter, and FLOP, and the DSP-SPPF model has a higher detection speed.

**Table 5:** Comparison of results of ablation experiments.

Optimal method	*m_AP__*0.5(%)	FPS(f/s)	Parameter(M)	FLOPs/G
NO	95.20	78.9234	37.620	106.472
DSP-backbone	95.69	79.9361	39.207	109.011
DSP-SPPF	95.63	**83.7978**	**32.115**	**102.064**
CANet	96.08	78.9315	37.630	106.479
DSP-YOLOv7-CA	**98.86**	80.5433	33.711	104.609

The bold font denotes which model performs best on a particular metric.

### 3.4 Comparison with other model results

**Figure 13:**
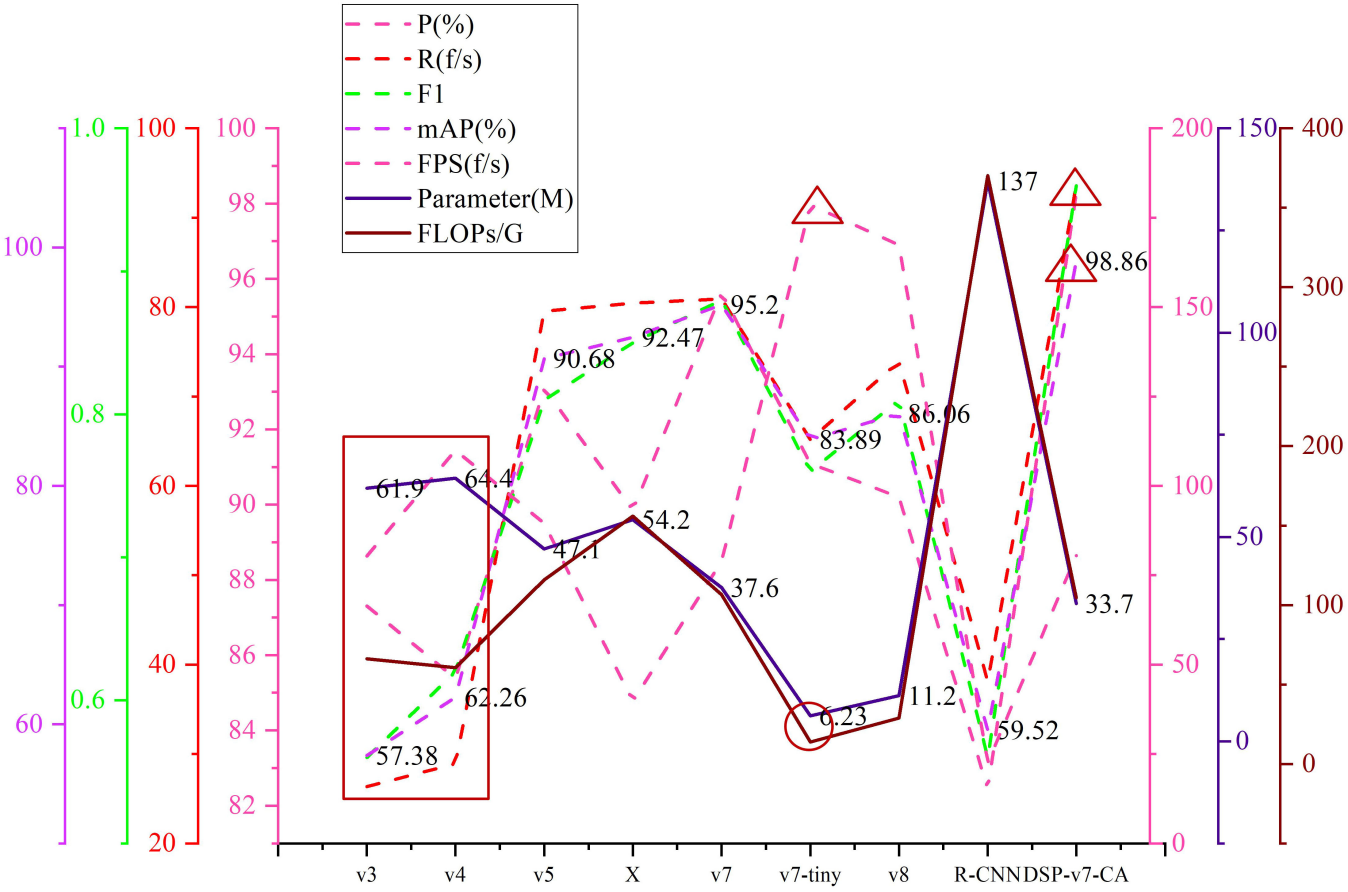
Performance comparison chart between the latest detection models.

To compare the performance between the models in this paper and the latest detection models, we conducted comparison tests, which include YOLOv3, YOLOv4, YOLOv5, YOLOX, YOLOv7-tiny, YOLOv8, Faster R-CNN, and DSP-YOLOv7-CA. The results are shown in **Table 6**, and the mAP value of the model in this paper is 98.86%, which is improved by 3.66 percentage points relative to YOLOv7 and 39.34 percentage points close to Faster R-CNN. Compared with YOLOv3, YOLOv4, YOLOv5, YOLOX, YOLOv7-tiny, and YOLOv8, the improvement is 41.48, 36.6, 8.18, 6.39, 14.97, and 12.8 percentage points, respectively. The memory footprint of the model in this paper is 33.711MB, which is 3.9M, 20.498M, and 13.346M less compared to YOLOv7, YOLOX, and YOLOv5, respectively. Compared to YOLOv3 and YOLOv4, the memory footprint is about half of them; compared to Faster R-CNN, it is one-fourth of them; compared to YOLOv8 compared to YOLOv8 and YOLOv7-tiny, the memory usage is increased by a factor of 3; compared to YOLOv7-tiny, the memory usage is increased by a factor of 5. DSP-YOLOv7CA is not as fast as YOLOv8 and YOLOv7-tiny, which are two lightweight models, but it still has a significant advantage in detection speed compared to YOLOv7 and other standard target detection models. In **Figure 13**, the red rectangles show the performance of YOLOv3 and YOLOv4, and the red circles and red triangles are the best performance points. The older YOLO models perform poorly on occluded cherry tomato detection, and DSP-YOLOv7-CA has the highest accuracy among the nine detection models and is the best means of solving the occluded cherry tomato detection problem.

**Table 6:** Comparison of detection performance among different models.

Model	Network structure	P(%)	R(f/s)	F1	*m_AP_ _*0.5(%)	FPS(f/s)	Parameter(M)	FLOPs/G
YOLOv3	Darknet53	87.31	26.36	0.56	57.38	80.3854	61.949	66.171
YOLOv4	CSPDarknet53 and SPP	85.38	28.91	0.62	62.26	109.9375	64.363	60.527
YOLOv5	CSPDarknet53 and SPPB	93.09	79.55	0.81	90.68	89.4606	47.057	115.918
YOLOX	Darknet53 and SPPB	89.85	80.45	0.85	92.47	39.6921	54.209	156.011
YOLOv7	MCB and SPPCSPC	95.63	80.92	0.88	95.20	78.9234	37.620	106.472
YOLOv7-tiny	MCB and SPPCSPC	91.09	65.17	0.76	83.89	**178.5684**	**6.227**	**13.860**
YOLOv8	C2f and SPPF	90.20	73.92	0.81	86.06	167.3426	11.167	28.817
Faster R-CNN	VGG16	82.45	38.12	0.56	59.52	22.6547	137.099	370.210
DSP-YOLOv7-CA	DSP- MSB and DSP-SPPF	**98.3**	**93.64**	**0.96**	**98.86**	80.5433	33.711	104.609

The bold font denotes which model performs best on a particular metric.

### 3.5 Comparison of model detection effects

**Figure 14:**
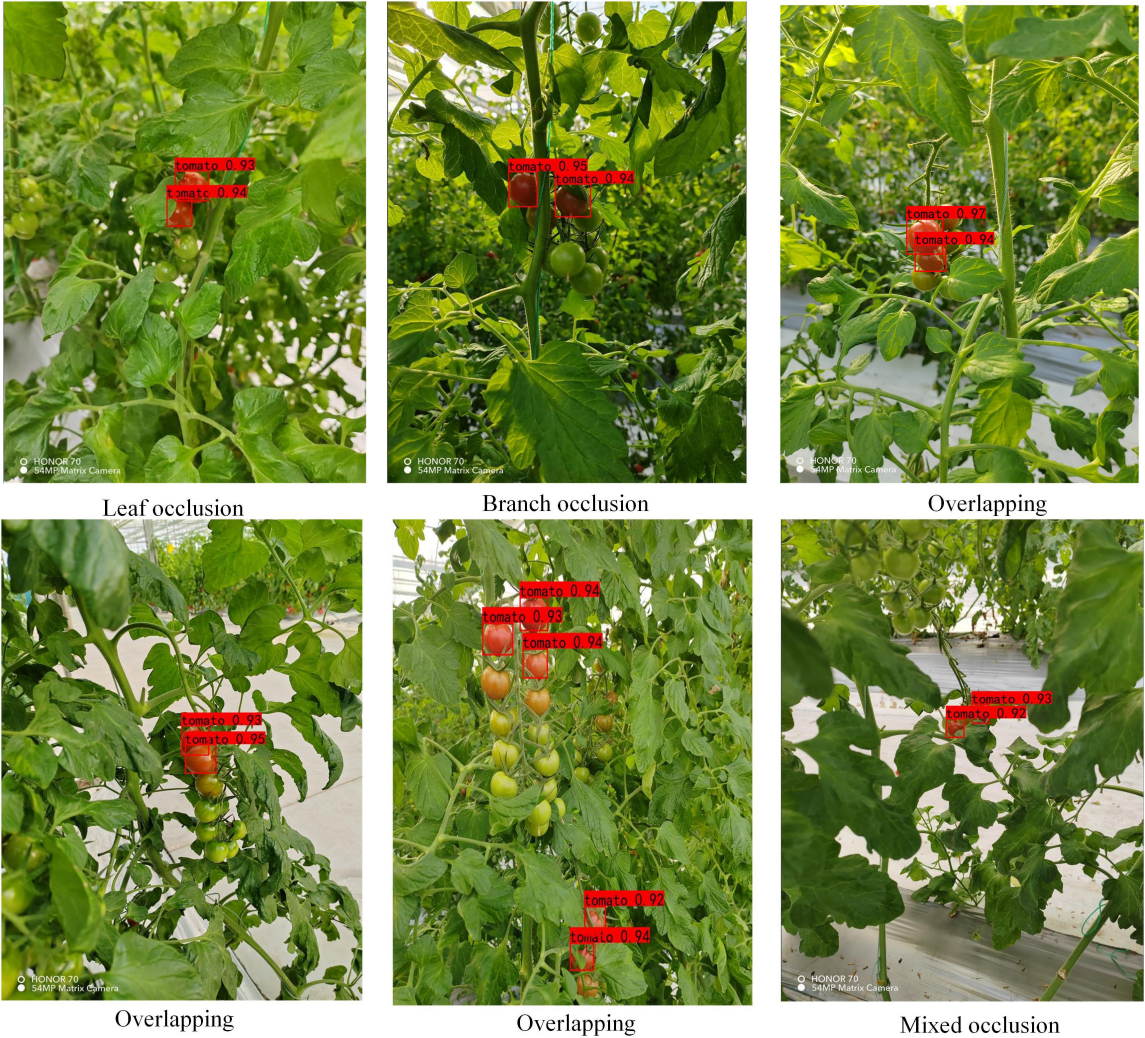
Detection of various light shade (0-30%).

To test the actual detection effect of the model in this paper, we look for cherry tomatoes in different occlusion situations in the dataset (TSOL), as shown in **Figure 14**, in light occlusion (0-30%), the six sets of maps tested include 15 ripe cherry tomato targets. The number of false detections is 0, the number of missed detections is 0, and the success rate is 100% for DSP-YOLOv7-CA. Due to the retention of a large amount of complete cherry tomato feature information, the detection effect is good.

**Figure 15:**
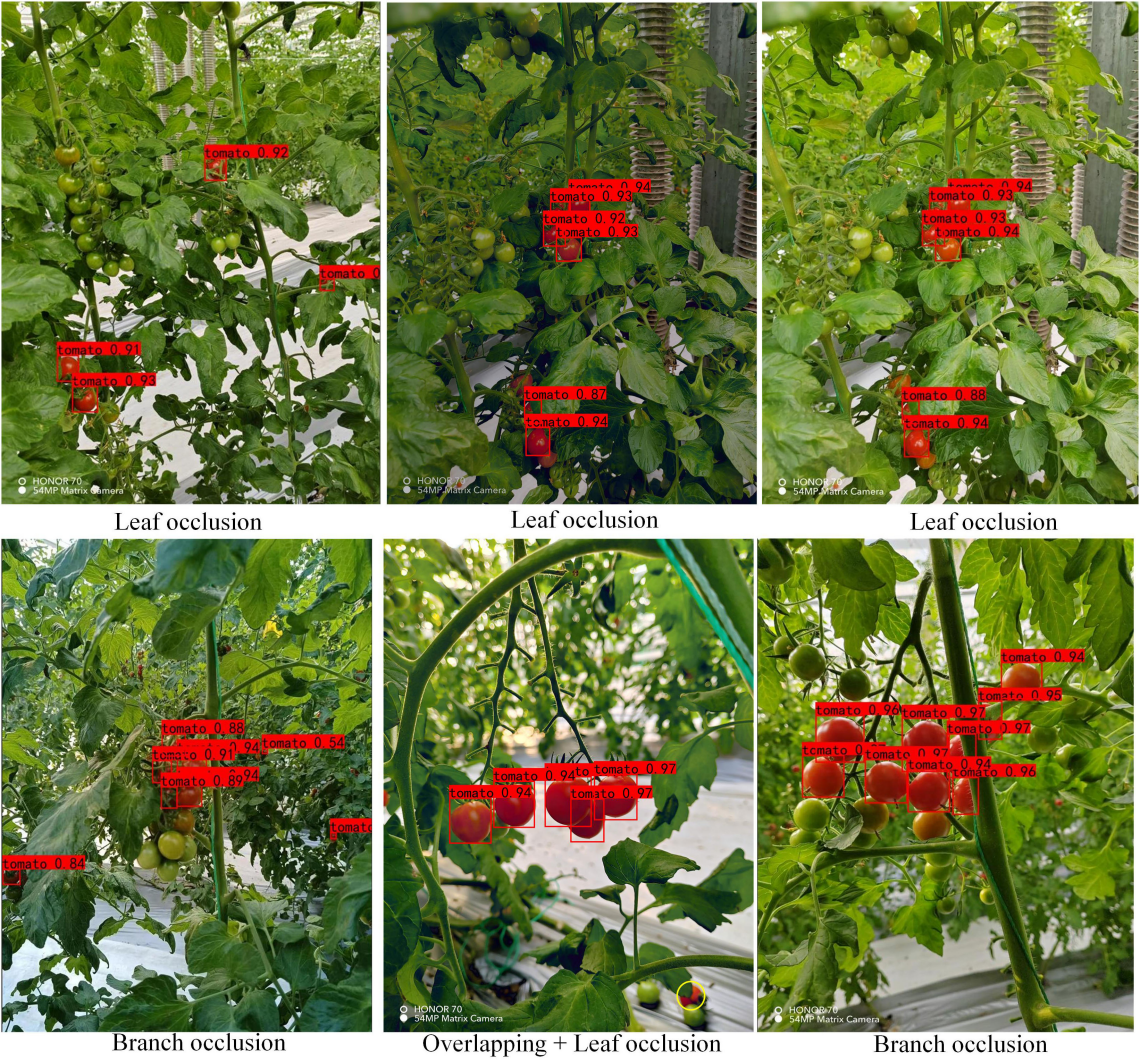
Detection of Various Moderate Occlusions (30-70%).

As shown in **Figure 15**, in moderate occlusion (30-70%), the six sets of images tested include 38 ripe cherry tomato targets; the number of false detections is 0, and the number of missed detections is 1. The detection success rate of the DSP-YOLOv7-CA is 97.4%. The missed cherry tomatoes are marked by yellow circles, obscured by leaves, relatively far away, and have fewer surface features, meeting the actual detection requirements.

This paper attempts to solve the problem of cherry tomato detection under different occlusions, and the cherry tomato detection under heavy occlusion (70-95%) better reflects the model’s performance. Therefore, this section compares the detection effect of DSP-YOLOv7-CA and the latest model YOLOv8, as shown in **Supplementary Figure 1**; the six sets of images tested include 51 ripe cherry tomato targets, and DSP-YOLOv7 -CA’s number of false detections is 0, the number of missed detections is 4, and the success rate is 92.2%, with a slight decrease in the success rate. The cherry tomatoes in the yellow box are long-distance small targets, which are not included in the test. It can be found that YOLOv8 has more missed detections relative to the model in this paper, and the missed targets are in a variety of occlusion situations. In contrast, at the edge of the picture, the detection effect is average due to the incompleteness of the cherry tomatoes.

As shown in **Supplementary Figure 2**, in severe occlusion (95-100%), six sets of images, including 28 severely occluded targets and 33 targets with another degree of occlusion, are tested, the number of false detections of DSP-YOLOv7-CA is 0, and the number of missed detections of severely occluded targets is 15. The success rate is 46.4%, which is a general effect of the detection, especially in the case of composite occlusion. Still, the degree of occlusion greater than 95% of the target is not considered as the detection category of this model.

To discuss the generalization ability of DSP-YOLOv7-CA to different occlusion scenes or environments other than cherry tomato, tests on other cherry tomato varieties, including ripe saint tomato, jade tomato, beautiful orange honey fragrance, and gold glittering tomato, were conducted in this section. The test results are shown in **Supplementary Figure 3**, where the Jade tomato has the highest confidence score due to the similarity in colour and shape between the Jade tomato and the dataset (TOSL) in this paper. In contrast, the Beautiful orange honey fragrance and Golden shiny tomato have lower confidence scores, and the ripe Beautiful orange honey fragrance and Golden shiny tomato showed a golden yellow colour, which was somewhat different from the dataset of this paper and appeared to be missed. Sage tomato presents a long strip shape, and this paper’s dataset has some differences; the detection of the location of the effect is general, this paper’s model has a certain degree of stability, and the future can update the dataset to achieve a variety of varieties of ripe small tomatoes recognition.

According to the positional relationship between cherry tomatoes and branches and leaves in **Figure 2**, this section builds a detection platform for different occlusion situations indoors through plastic models of cherry tomatoes and potted picking plants, with other branches passing through the cherry tomato model to realize branch occlusion, leaf occlusion through different sizes of leaf occlusion of cherry tomato models, and fruit overlap through the stacking of two cherry tomato models. The detection results are shown in **Supplementary Figure 4**; DSP-YOLOv7-CA can not accurately detect cherry tomatoes with 95-100% complex shading area, and in the case of overlapping each other, there are many times of missed detection, and the detection success rate of DSP-YOLOv7-CA for cherry tomatoes with shading area less than 95% is 94.7%.

Therefore, synthesizing the above analysis results, it can be concluded that the actual detection effect of DSP-YOLOv7-CA on cherry tomatoes with less than 70% shading in the dataset (TSOL) is better; there will be a leakage in the detection of cherry tomatoes with 70-95% shading, and the effect is general in the detection of cherry tomatoes with higher than 95%, and the different cherry tomato varieties and cherry tomato models with the dataset differ significantly, the model can not accurately recognize the feature information, and the detection effect is general. However, DSP-YOLOv7-CA significantly outperforms the latest YOLOv8 detection model in terms of performance.DSP-YOLOv7-CA balances detection speed and accuracy in shading cherry tomato picking ensures a low false detection rate, improves detection speed simultaneously, and is more suitable for cherry tomato picking.

## 4 Discussion

In this paper, the DSP-YOLOv7-CA model focuses more on the feature information around the target and has good detection ability in the face of occluded cherry tomatoes in the natural environment. However, there are still potential drawbacks, such as the relatively average detection effect in the case of severe occlusion, the average detection effect in the face of different varieties of cherry tomatoes, and the fact that it is limited to cherry tomatoes similar to the dataset in shape and colour. In the future, different types of cherry tomatoes can be added to enrich the dataset of cherry tomatoes. In this paper, cherry tomatoes with masked area higher than 95% are not used as the detection target; in the future, the model should deal with more complex masking scenarios, such as cherry tomatoes with masked area higher than 95%; this model can learn the detection method of the significant model Segment anything, which is effectively queried by various input hints, corresponding to fuzzy suggestions for multiple objects, and then outputs multiple effective masks and associated confidence scores Kirillov et al. (2023). By designing more advanced datasets and detection models, the detection of various occluded targets in natural environments is realized.

## 5 Conclusion

The dense overlapping of cherry tomatoes and the occlusion situation of leaves and branches are common phenomena in the natural environment, and solving the complex problem of occluded cherry tomato detection will improve the efficiency of cherry tomato picking robots. This paper proposes an occluded cherry tomato detection model DSP-YOLOv7-CA with good performance. First, cherry tomato images with different degrees of occlusion are collected, four occlusion areas and four occlusion methods are defined, and a cherry tomato data set (TOSL) is constructed. Then, the deep residual DSP-MultiBlock module with multiscale detection was used in the backbone network, and the detection accuracy reached 95.69%, which was improved by 0.49 percentage points compared with the original model. Then, using both the deep residual DSP-Multiblock module with multiscale detection and the DSP-SPPF module, the average accuracy of DSP-YOLOv7 is improved by almost one percentage point compared to the original model, 3.3 f/s enhance the FPS, the amount of parameters is reduced by 4M, and the floating-point computation is reduced by 1.8s/G. Introducing coordinates at critical locations in the enhanced feature extraction network Attention Mechanism (CA) layer improves the model’s accuracy by 0.88 percentage points. Then, the DSP-YOLOv7-CA model is obtained by combining the individual best methods, and the AP value of the model reaches 98.86%, which is improved by 3.66 percentage points concerning YOLOv7, 39.34 percentage points concerning Faster R-CNN, and 12.8 percentage points concerning the latest target detection model YOLOv8. In the actual detection, DSP-YOLOv7-CA has a better detection effect on cherry tomatoes with less than 70% occlusion, misses in the detection of cherry tomatoes with 70-95% occlusion, and has an average impact on the detection of cherry tomatoes with higher than 95% occlusion, which is better than the latest target detection model YOLOv8. This model can satisfy the picking while maintaining the detection accuracy. Robot’s real-time needs while maintaining detection accuracy.

## Data availability statement

The raw data supporting the conclusions of this article will be made available by the authors, without undue reservation.

## Author contributions

GH: Methodology, Validation, Writing – original draft, Writing – review & editing. HC: Methodology, Writing – review & editing. YM: Methodology, Writing – review & editing. MJ: Data curation, Writing – review & editing. CH: Data curation, Writing – review & editing. CJ: Validation, Writing – review & editing. RN: Data curation, Writing – review & editing.

## References

[B1] Chen S. Zou X. Zhou X. Xiang Y. Wu M. Zhang J. (2023). Study on fusion clustering and improved yolov5 algorithm based on multiple occlusion of camellia oleifera fruit. Comput. Electron. Agric. 206, 107706. doi: 10.1016/j.compag.2023.107706

[B2] FAO (2018). Food and Agriculture Organization of the United Nations (Rome: CAB International).

[B3] Feng Q. Cheng W. Li Y. Wang B. Chen L. (2022). Tomato plant pruning point localization method based on mask r-cnn. Trans. Chin. Soc. Agric. Eng. 38, 128–135. doi: 10.11975/j.issn.1002-6819.2022.03.015

[B4] Feng Q. Cheng W. Zhang W. (2021). “Visual Tracking Method of Tomato Plant Main-Stems for Robotic Harvesting,” in 2021 IEEE 11th Annual International Conference on CYBER Technology in Automation, Control, and Intelligent Systems (CYBER). (Jiaxing, China: IEEE) 886–890. doi: 10.1109/CYBER53097.2021.9588275

[B5] Gai R. Li M. Wang Z. Hu L. Li X. (2023). Yolov5s-cherry: Cherry target detection in dense scenes based on improved yolov5s algorithm. J. Circuits Systems Comput. 32, 2350206. doi: 10.1142/S0218126623502067

[B6] He B. Zhang Y. Gong J. (2022). A tomato fruit rapid identification system in night greenhouse based on improved yolov5 and icnet. Trans. Chin. Soc. Agric. Machinery 53, 201–208. doi: 10.6041/j.issn.1000-1298.2022.05.020

[B7] He K. Zhang X. Ren S. Sun J. (2016). “Deep residual learning for image recognition,” in 2016 IEEE Conference on Computer Vision and Pattern Recognition (CVPR). (Las Vegas, USA). 770–778. doi: 10.1109/CVPR.2016.90

[B8] Hou Q. Zhou D. Feng J. (2021). “Coordinate attention for efficient mobile network design,” in Proceedings of the IEEE/CVF conference on computer vision and pattern recognition. 13713–13722.

[B9] Khan A. Hassan T. Shafay M. (2023). “Convolutional transformer for autonomous recognition and grading of tomatoes under various lighting, occlusion, and ripeness conditions,” in The title of the conference proceedings (arXiv), arXiv:2306.10921.

[B10] Kirillov A. Mintun E. Ravi N. (2023). “Segment anything,” in The title of the conference proceedings (arXiv).

[B11] Li W. Zhang Y. Mo J. (2020). Field pedestrian and agricultural machinery obstacle detection based on improved yolov3-tiny. Trans. Chin. Soc. Agric. Machinery 51, 1–8+33. doi: 10.6041/j.issn.1000-1298.2020.S1.001

[B12] Liu J. He J. Chen H. (2023). Tomato cluster detection model based on improved yolov4 and icnet. Trans. Chin. Soc. Agric. Machinery, 1–10.

[B13] Lv Z. Zhang F. Wei X. (2023). Tomato flower and fruit recognition in greenhouse using combined enhanced yolox-vit method. Trans. Chin. Soc. Agric. Eng. 39, 124–134. doi: 10.11975/j.issn.1002-6819.202211246

[B14] Mbouembe P. Liu G. Sikati J. Kim S. Kim J. (2023). An efficient tomato-detection method based on improved yolov4-tiny model in complex environment. Front. Plant Sci. 14. doi: 10.3389/fpls.2023.1150958 PMC10106724 37077640

[B15] Sa I. Ge Z. Dayoub F. Upcroft B. Perez T. McCool C. (2016). Deepfruits: A fruit detection system using deep neural networks. Sensors 16, 1222. doi: 10.3390/s16081222 27527168 PMC5017387

[B16] Saedi S. Khosravi H. (2020). A deep neural network approach towards real-time onbranch fruit recognition for precision horticulture. Expert Syst. Appl. 159, 113594. doi: 10.1016/j.eswa.2020.113594

[B17] Sandler M. Howard A. Zhu M. (2018). “Mobilenetv2: Inverted residuals and linear bottlenecks,” in Proceedings of the IEEE conference on computer vision and pattern recognition. 4510–4520.

[B18] Su F. Zhao Y. Wang G. (2022). Tomato maturity classification based on se-yolov3-mobilenetv1 network under nature greenhouse environment. Agronomy 12, 1638. doi: 10.3390/agronomy12071638

[B19] Wang C. Y. Bochkovskiy A. Liao H. Y. M. (2023). “Yolov7: Trainable bag-of-freebies sets new state-of-the-art for real-time object detectors,” in Proceedings of the IEEE/CVF Conference on Computer Vision and Pattern Recognition. 7464–7475.

[B20] Wang X. Long J. (2023). “Citrus detection application of based on improved yolov3 algorithm,” in Third International Conference on Machine Learning and Computer Application (ICMLCA) (SPIE), Vol. 12636. 280–286. doi: 10.1117/12.2675534

[B21] Wang Z. Zan Y. Ling Y. Wang X. Meng D. Nie L.. (2022). An improved faster r-cnn model for multi-object tomato maturity detection in complex scenarios. Ecol. Inf. 72, 101886. doi: 10.1016/j.ecoinf.2022.101886

[B22] Xu Y. (2013). Two-stage approach for detecting slightly overlapping strawberries using hog descriptor. Biosyst. Eng. 115, 144–153. doi: 10.1016/j.biosystemseng.2013.03.011

[B23] Yan J. Wang P. Wang T. (2021). “Identification and localization of optimal picking point for truss tomato based on mask r-cnn and depth threshold segmentation,” in 2021 IEEE 11th Annual International Conference on CYBER Technology in Automation, Control, and Intelligent Systems (CYBER). (IEEE) 899–903. doi: 10.1109/CYBER53097.2021.9588274

[B24] Yang C. Liu Y. Wang Y. Xiong L. Xu H. Zhao W. (2019). Research on recognition and positioning system of citrus harvesting robot in natural environment. Trans. Chin. Soc. Agric. Machinery 50, 14–22+72. doi: 10.6041/j.issn.1000-1298.2019.12.002

[B25] Yang J. Qian Z. Zhang Y. (2022). Real-time tomato recognition in complex environments using improved yolov4-tiny. Trans. Chin. Soc. Agric. Eng. 38, 215–221. doi: 10.11975/j.issn.1002-6819.2022.09.023

[B26] Yu Y. Zhang K. Yang L. (2019). Fruit detection for strawberry harvesting robot in non-structural environment based on mask r-cnn. Comput. Electron. Agric. 163, 104846. doi: 10.1016/j.compag.2019.06.001

[B27] Yuan T. Lv L. Zhang F. (2020). Robust cherry tomatoes detection algorithm in greenhouse scene based on ssd. Agriculture 10, 160. doi: 10.3390/agriculture10050160

[B28] Zhang J. (2014). Analysis on the international competitiveness of chinese clothing industry. Appl. Mechanics Materials 685, 697–701. doi: 10.4028/www.scientific.net/AMM.685.697

[B29] Zhang J. Bi Z. Yan Y. (2023b). Fast tomato recognition in greenhouse based on attention mechanism and improved yolo. Trans. Chin. Soc. Agric. Machinery 54, 236–243. doi: 10.6041/j.issn.1000-1298.2023.05.024

[B30] Zhang F. Chen Z. Ali S. (2023a). Multi-class detection of cherry tomatoes using improved yolov4tiny. Int. J. Agric. Biol. Eng. 16, 225–231. doi: 10.25165/j.ijabe.20231602.7744

[B31] Zhang Q. Chen J. Li B. Xu C. (2021). Recognition and localization of tomato cluster picking points based on rgb-d information fusion and object detection. Trans. Chin. Soc. Agric. Eng. 37, 143–152. doi: 10.11975/j.issn.1002-6819.2021.18.017

[B32] Zhang F. Lv Z. Zhang H. Guo J. Wang J. Lu T.. (2022). Verification of improved yolox model in detection of greenhouse crop organs: Considering tomato as example. Comput. Electron. Agric. 205, 107582. doi: 10.1016/j.compag.2022.107582

[B33] Zheng T. Jiang M. Feng M. (2021). Survey on visual-based object recognition and localization methods for harvesting robots. J. Instrumentation Measurement 42, 28–51. doi: 10.19650/j.cnki.cjsi.J2107650

[B34] Zheng T. Jiang M. Li Y. (2022). Research on tomato detection in natural environment based on rc-yolov4. Comput. Electron. Agric. 198, 107029. doi: 10.1016/j.compag.2022.107029

