# Supplementary figures and images for: An occluded cherry tomato recognition model based on improved YOLOv7
Source: Front Plant Sci. 2023 Oct 20;14:1260808. doi: 10.3389/fpls.2023.1260808 (PMC10625446; doi:10.3389/fpls.2023.1260808)

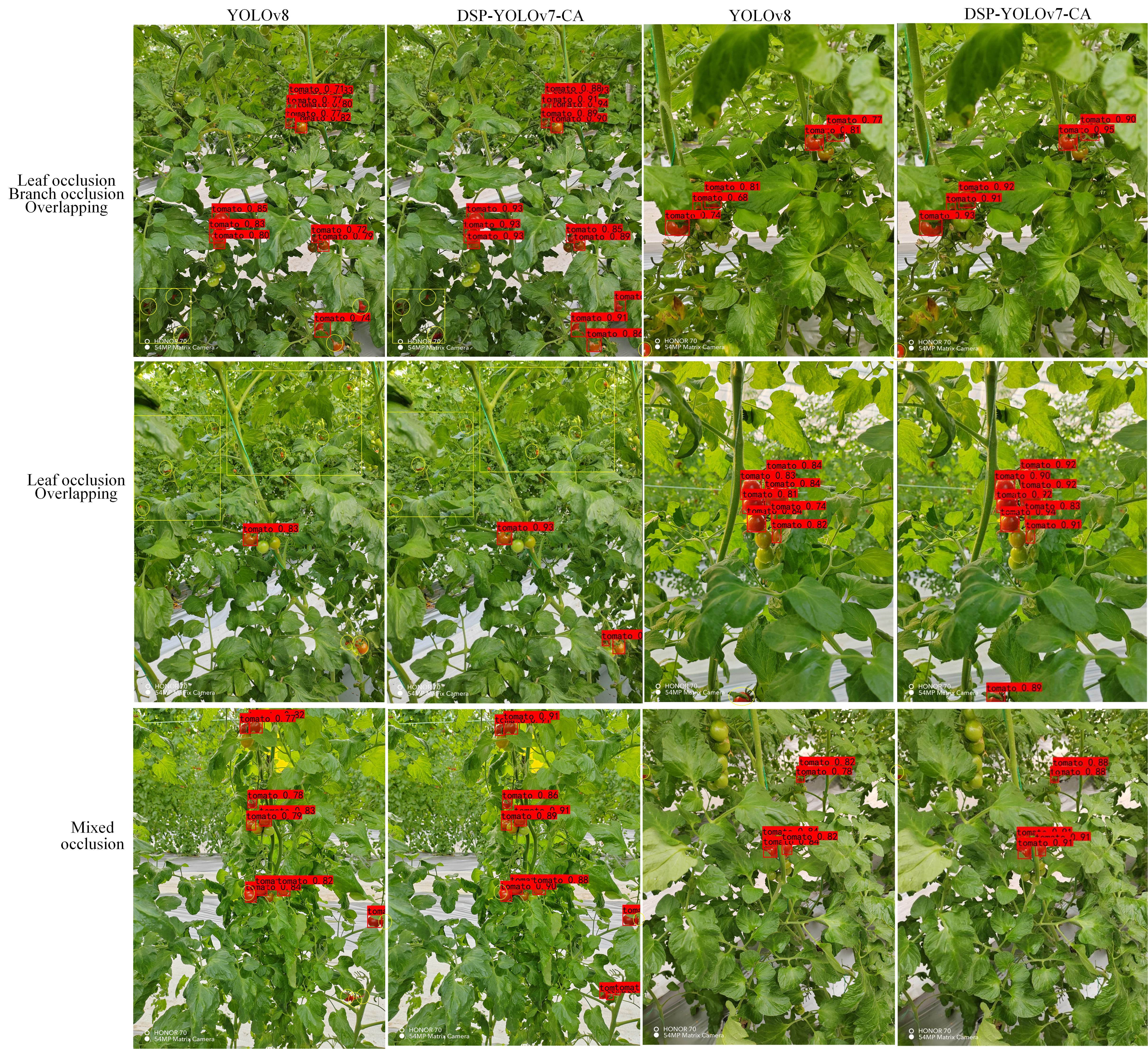

Supplement: Supplementary file 1 [file Image_1.jpeg]

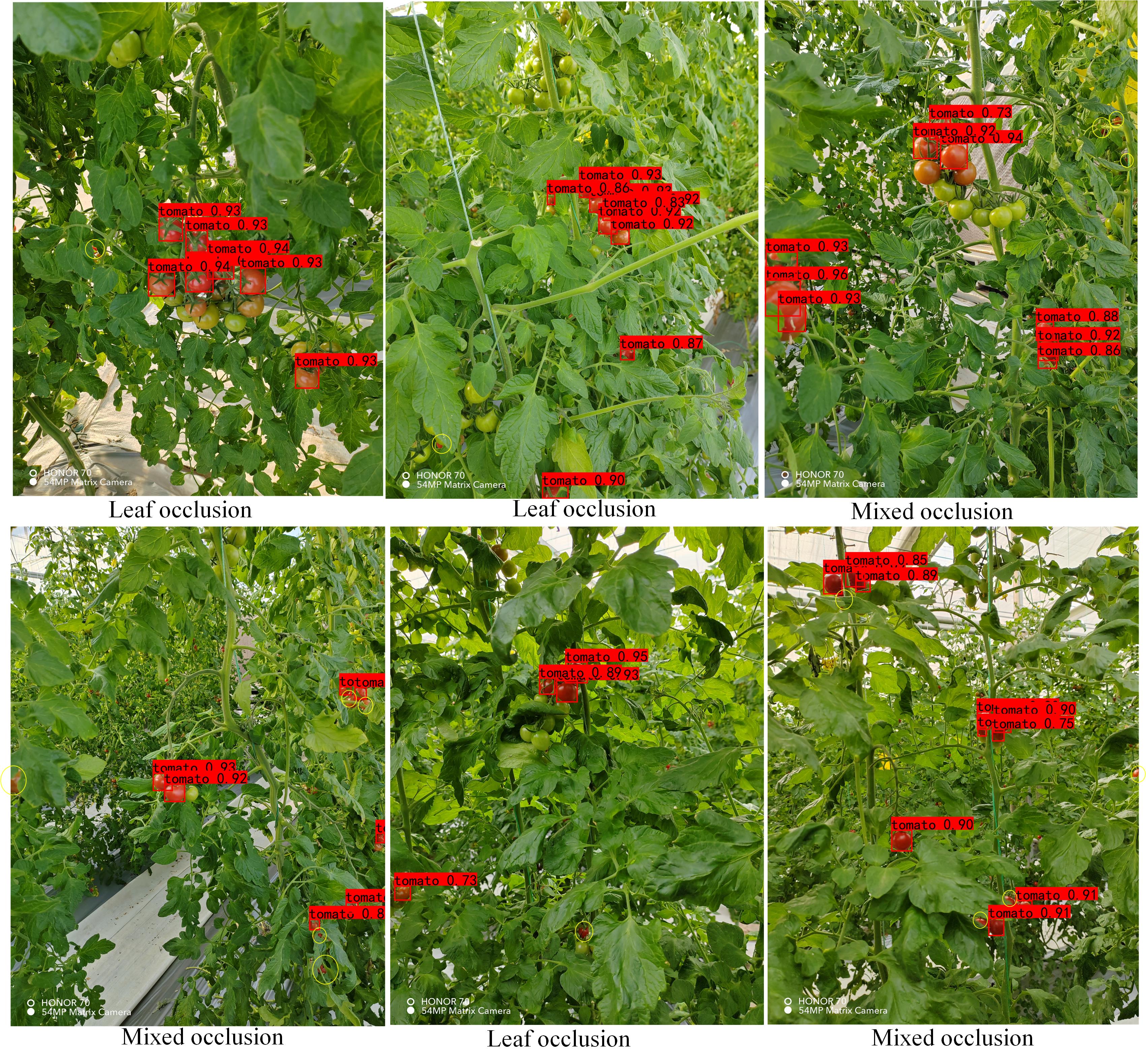

Supplement: Supplementary file 2 [file Image_2.jpeg]

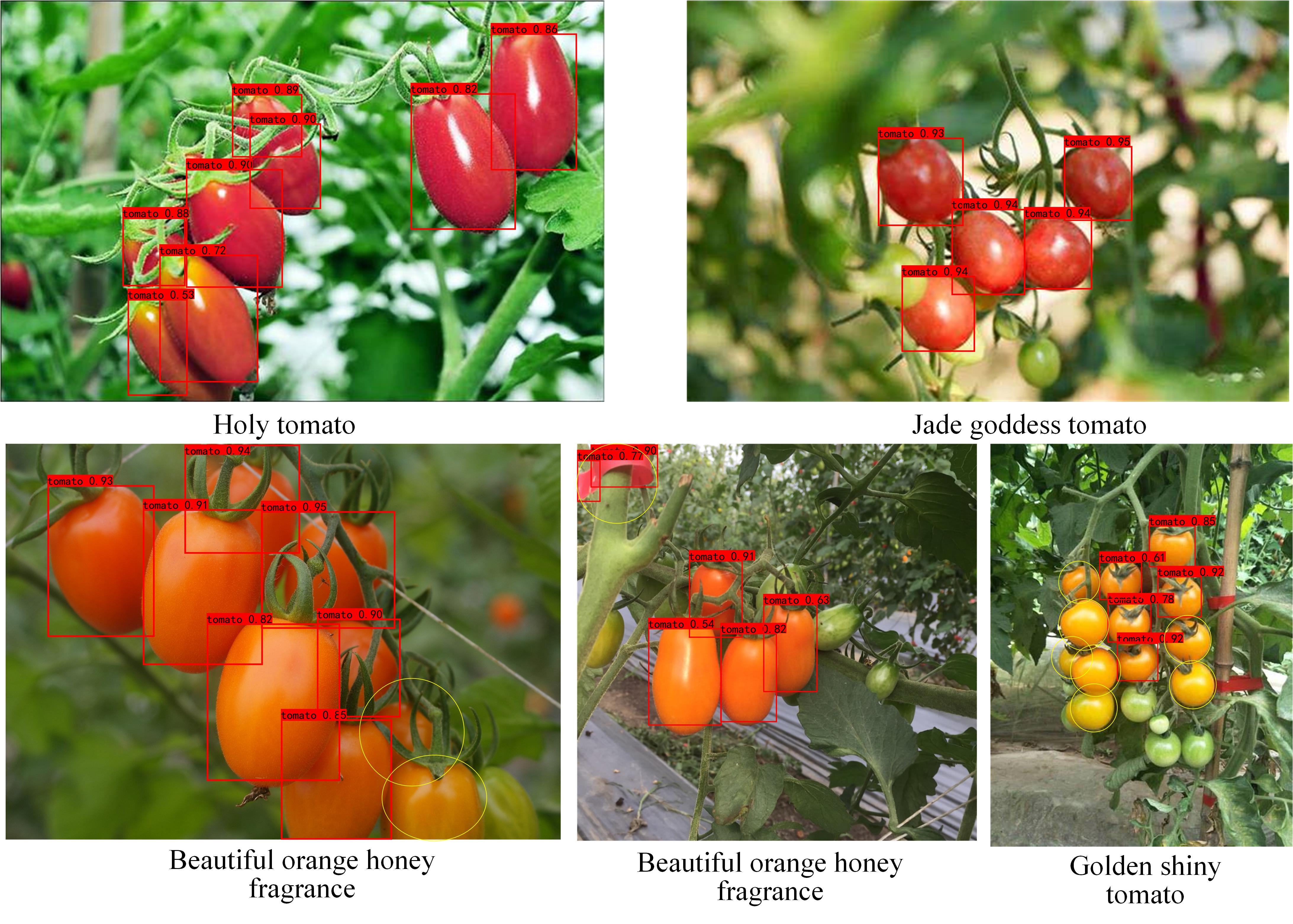

Supplement: Supplementary file 3 [file Image_3.jpeg]

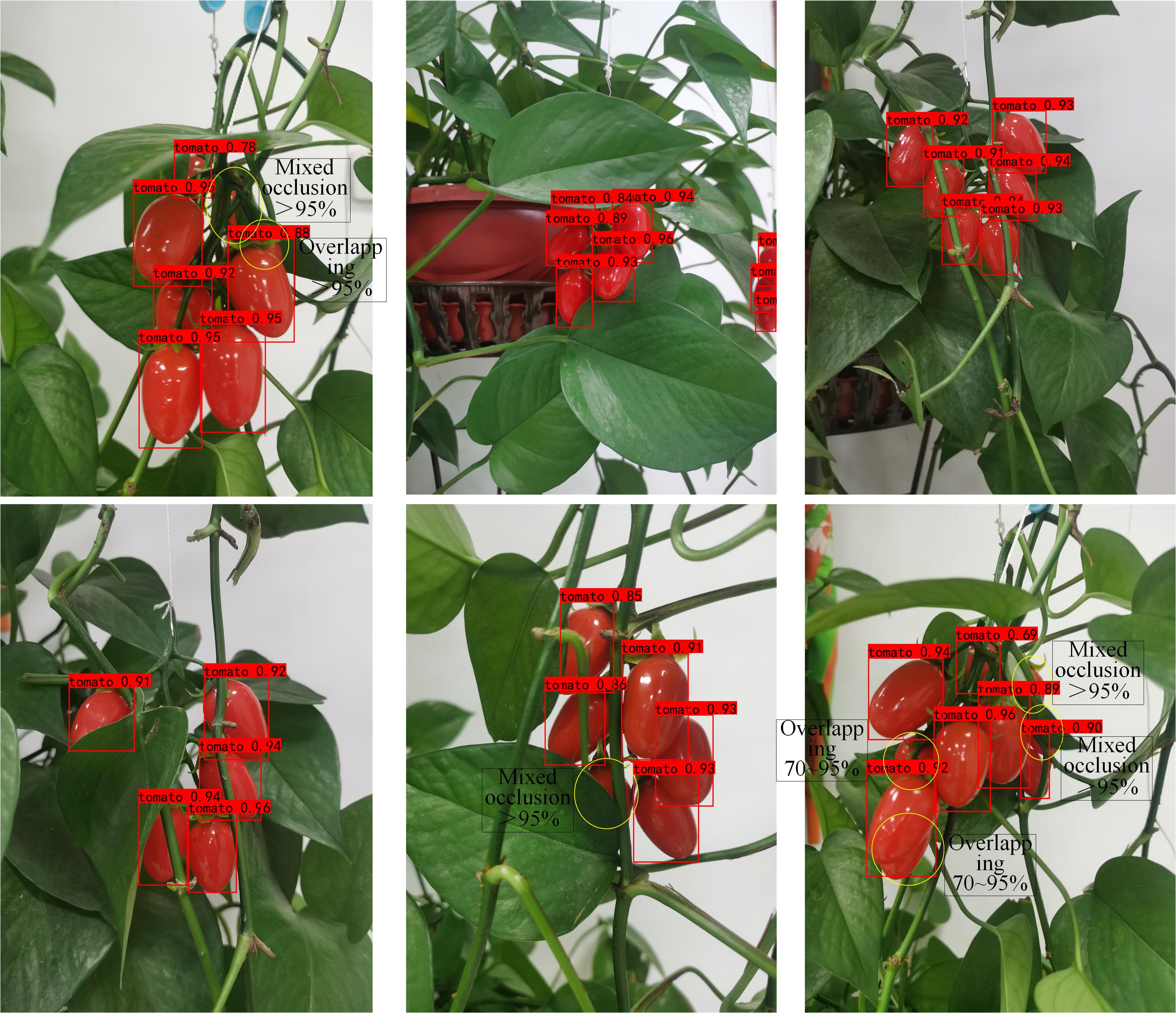

Supplement: Supplementary file 4 [file Image_4.jpeg]
